# Development and Implementation of a Core Training Protocol: Effects on Muscle Activation, Hypertrophy, Balance, and Quality of Life in Recreationally Active Adults

**DOI:** 10.3390/mps8040077

**Published:** 2025-07-08

**Authors:** Ioannis Tsartsapakis, Aglaia Zafeiroudi, Ioannis Trigonis, Christos Lyrtzis, Konstantinos Astrapellos

**Affiliations:** 1 Department of Physical Education and Sport Sciences at Serres, Aristotle University of Thessaloniki, 62100 Serres, Greece; 2 Department Physical Education and Sport Science, University of Thessaly, 42100 Trikala, Greece; azafeiroudi@uth.gr; 3 Department of Physical Education and Sport Science, Democritus University of Thrace, 69100 Komotini, Greece; itrigon@phyed.duth.gr (I.T.); kastrape@phyed.duth.gr (K.A.); 4 Department of Anatomy and Surgical Anatomy, Aristotle University of Thessaloniki, 54124 Thessaloniki, Greece; lyrtzischristos@gmail.com

**Keywords:** recreational exercisers, overhead medicine ball slam, motion analysis, gym-based exercise, leisure fitness training, health club exercisers, trunk stabilization, neuromuscular efficiency, electromyography, postural control

## Abstract

Core stability is fundamental to posture, balance, and force transmission throughout the kinetic chain. Although traditionally associated with athletic performance, emerging research highlights its broader applicability to recreational fitness. This study investigates the effects of an eight-week core training program on muscle hypertrophy, static balance, and neuromuscular control in recreationally active, non-athletic adults. Participants will undertake a structured intervention comprising progressive triads targeting core stability, strength, and power. Assessment methods include surface electromyography (EMG), ultrasound imaging, three-dimensional force plates, Kinovea motion analysis, and the Satisfaction With Life Scale (SWLS) questionnaire. Expected outcomes include enhanced core muscle activation, improved static balance, and increased core-generated force during overhead medicine ball slam trials. Additionally, the intervention aims to facilitate hypertrophy of the transverse abdominis, internal oblique, and lumbar multifidus muscles, contributing to spinal resilience and motor control. This protocol bridges gaps in core training methodologies and advances their scalability for recreational populations. The proposed model offers a structured, evidence-informed framework for improving core activation, postural stability, muscle adaptation, movement efficiency, and perceived quality of life in recreationally active individuals.

## 1. Introduction

Core stability is a fundamental component of human movement, forming the basis for effective posture, balance, and force transmission throughout the kinetic chain [1]. A wealth of research underscores the pivotal role of the core musculature in mitigating injury risk and enhancing physical performance. These findings highlight the need for targeted interventions aimed at improving strength, endurance, and neuromuscular control [2,3,4].

Beyond athletic benefits, core stability serves as a key public health component, contributing to injury prevention, musculoskeletal resilience, and long-term movement efficiency. By enhancing neuromuscular engagement and postural endurance, structured core training protocols offer accessible solutions for mitigating the rising prevalence of sedentary-related dysfunctions in the general population. The core is widely understood as an integrated system comprising the passive spinal column (bony and connective structures), active musculature, thoracolumbar fascia, and neural control mechanisms [5,6]. The musculature itself includes the back, abdominal, pelvic floor, diaphragm, hip, and gluteal muscles, which collectively stabilize the spine, facilitate functional movement, and transfer forces between the upper and lower extremities [7,8]. Akuthota and Nadler [9] conceptualized the core as a three-dimensional structure, likened to a box with the diaphragm forming the roof, the pelvic floor the base, and the abdominal and dorsal muscles comprising the walls. While traditionally focused on the lumbar spine and pelvis, more recent models, such as Kolar et al.’s [10] Integrated Spinal Stabilization System (ISSS), emphasize a holistic approach by incorporating the cervical spine alongside deep cervical flexors, spinal extensors, and core musculature of the thoracic and lumbar regions. This expanded view reflects the increasingly recognized role of the core in maintaining both static and dynamic stability across the entire spinal column.

Beyond stabilization, the core serves as a critical hub for force transfer, enabling efficient and coordinated limb movements. As McGill [11] explains, the core functions primarily as a stabilizer and force conduit rather than a prime mover. Completing, Malanga et al. [12] describe its dual role as a stabilizer and a force generator during limb initiation. These complementary perspectives underscore the centrality of the core in the human motor chain, as highlighted by Hoogenboom and Kiesel [13].

Despite its importance, scientific debate persists regarding which muscles serve as primary stabilizers and how they should be prioritized during core training. Some models distinguish between “local” and “global” muscles [14,15], while others emphasize the coordinated activation of all trunk muscles as task-dependent [16,17]. Contemporary approaches frequently categorize core muscles into stabilizing muscles and movement-producing muscles, underscoring the critical role of neuromuscular control in dynamic stabilization [18]. Stability, therefore, is less a product of strength alone and more reliant on endurance and motor control patterns, as insufficient endurance or control—rather than strength—may compromise stability in both everyday and athletic activities [19,20].

Integrated core training programs must address this complexity, balancing the demands of strength, endurance, and neuromuscular coordination. This study seeks to explore these elements through a structured intervention program designed to evaluate the effects of core training on muscle hypertrophy, static balance, and functional motor control.

### 1.1. Core Training in Recreational Exercisers

Core training methodologies have progressed significantly, shifting from traditional static exercises to dynamic and functional protocols aimed at optimizing neuromuscular coordination, force transmission, and movement efficiency across diverse populations [4,21,22,23,24,25,26,27,28,29,30,31]. While initial core interventions primarily targeted athletic performance enhancement, recent research emphasizes their applicability for recreational fitness, providing benefits such as postural stabilization, movement precision, and injury prevention [32,33,34].

Rodríguez-Perea et al. [32] demonstrated that core-specific protocols significantly enhance postural stability, throwing velocity, and jump mechanics, reinforcing the utility of structured core training for general fitness practitioners. Similarly, Tallent et al. [33] found that neuromuscular adaptations from core exercises improve intermuscular coordination and corticospinal excitability, supporting their role in movement efficiency and injury resilience in previously inactive individuals. Zemková & Zapletalová [34] further emphasized that core stability training enhances spinal health, back muscle endurance, and functional movement, making it highly relevant for recreational fitness populations.

### 1.2. Transitioning from Athlete-Focused Protocols to Recreational Applications

Despite the extensive research conducted on core training in athletic contexts, its principles can be effectively adapted for non-athlete individuals seeking to enhance spinal stability, movement control, and long-term physical well-being [4,22,23,24,25,26,27,28]. Contrary to the high-intensity, sport-specific programming that is customary for elite athletes, recreational individuals stand to benefit from scalable core training strategies that emphasize gradual progression, postural endurance, and movement efficiency [35]. Such scalable strategies align with public health initiatives, as they offer accessible and adaptable solutions for injury prevention, workplace wellness, and movement rehabilitation, benefiting diverse populations beyond competitive athletes.

Research has demonstrated that dynamic and functional core exercises, which were initially developed for sports applications, can be equally beneficial for general fitness when appropriately scaled and structured. Core training strategies, which include stability-focused drills, progressive strength exercises, and controlled power movements, have been shown to enhance coordination, neuromuscular control, and posture alignment. These strategies can promote injury prevention and movement quality without excessive physical strain. [32,33,34].

Additionally, while athletic protocols prioritize peak power output, recreational fitness adaptations emphasize functional movement training, ensuring long-term accessibility and adherence. The integration of bodyweight exercises, resistance-based stability drills, and progressively loaded core exercises has been demonstrated to enhance core endurance and structural stability. This, in turn, has been shown to reinforce movement efficiency and musculoskeletal resilience [36].

Core training differs from traditional abdominal and back strengthening approaches in both scope and function. While conventional methods often isolate superficial muscles (e.g., rectus abdominis, erector spinae) through repetitive flexion or extension, core training emphasizes integrated, multi-planar control of the trunk and pelvis. This distinction is particularly relevant in sports requiring rotational power, postural endurance, and dynamic stabilization—such as tennis, golf, martial arts, and handball—where core-based strategies have demonstrated superior transfer to performance and injury prevention [37,38].

### 1.3. Core Training as a Foundational Component of General Fitness

Core stability plays a critical role in human movement, contributing to postural alignment, neuromuscular engagement, and effective force transmission throughout the kinetic chain [6,7,8,9,10,11]. Research confirms that structured core training reduces injury susceptibility, enhances dynamic balance, and improves spinal integrity, making it an essential component of recreational fitness programs. These benefits extend far beyond individual fitness, directly supporting public health strategies focused on reducing musculoskeletal disorders, optimizing movement efficiency, and promoting lifelong functional mobility across diverse populations.

Rodríguez-Perea et al. [32] and Tallent et al. [33] emphasize that structured core interventions enhance deep-core muscle activation, thereby reinforcing motor control strategies that mitigate compensatory movement patterns and augment spinal durability. Additionally, Zemková & Zapletalová [34] emphasize that progressive core stability training optimizes postural endurance, ensuring long-term movement efficiency for general fitness practitioners. Furthermore, the core training method has been associated with various neuromuscular adaptations, which, according to recent literature [33,34], may include improved motor unit recruitment, greater intermuscular coordination, and enhancements in corticospinal excitability. It is hypothesized that these mechanisms facilitate more efficient movement execution, thereby potentially underpinning the observed improvements in strength, balance, and postural control following core-specific interventions.

The findings, when considered collectively, support the broad applicability of core training beyond the context of competitive sports, serving as a critical intervention for movement precision, injury prevention, and functional mobility. The implementation of structured, scalable protocols is instrumental in ensuring accessibility for non-athletes, thereby reinforcing neuromuscular coordination, postural resilience, and quality of life improvements across diverse fitness levels.

The objective of this study is to design, implement, and evaluate a structured eight-week core training protocol intended to enhance core muscle activation, postural control, deep-core hypertrophy, movement efficiency, and perceived quality of life in recreationally active adults. The protocol has been developed with the objective of enhancing neuromuscular activation, postural stability, muscle hypertrophy, and functional movement efficiency in healthy, active adults. The protocol under consideration integrates progressive core-specific exercises with the objective of optimizing core muscle engagement. It is hypothesized that this will result in enhanced spinal integrity, improved kinetic chain coordination, and elevated athletic performance metrics. Therefore, the scientific hypothesis of the study is as follows:

(1) The eight-week core training program will enhance the activation of the rectus abdominis, the external oblique abdominis, and the iliocostalis lumborum muscles.
(2) The eight-week core training program will enhance core-generated force output as measured by the medicine ball slam exercise on a force plate.
(3) The eight-week core training program will help improve the static balance of the participants.
(4) The eight-week core training program will enhance the muscle thickness of the transverse abdominis, internal oblique abdominis, and lumbar multifidus, as assessed via ultrasound imaging.
(5) The eight-week core training program will enhance torso and arm angular velocity during the medicine ball overhead slam, as measured through biomechanical analysis using Kinovea software.

## 2. Experimental Design

### 2.1. Trial Design

This study has been prospectively registered on ClinicalTrials.gov under the identifier AUTH-CORE-STUDY NCT07025395, ensuring transparency and alignment with international standards for interventional research protocols.

The present study protocol has also been developed and reported in accordance with the SPIRIT 2013 Statement (Standard Protocol Items: Recommendations for Interventional Trials) [39], which provides guidance for the essential content of clinical trial protocols. Furthermore, the protocol adheres to the Template for Intervention Description and Replication (TIDieR) checklist and guide [40] and the Consensus on Exercise Reporting Template (CERT) [41], ensuring comprehensive, transparent, and replicable documentation of the core training intervention.

All completed checklists (SPIRIT, TIDieR, CERT) and the ClinicalTrials.gov registration certificate are available to reviewers and readers in Supplementary File S2. The official approval letter from the Ethics Committee is provided separately in Supplementary File S3.

This study employs a two-parallel group randomized controlled trial (RCT) design. The experimental group will undergo structured core training, while the control group will continue regular physical activity without targeted interventions. Detailed descriptions of exercise parameters, progression models, training environment, and measurement techniques are provided to enhance transparency and reproducibility.

The study protocol has been reviewed and approved by the Local Ethics Research Committee of the School of Physical Education and Sport Science at Serres, Aristotle University of Thessaloniki (Approval Code: ERC-013/2025, issued on 11 March 2025), in accordance with the University’s Code of Ethics for Research Involving Human Participants, the principles of the Declaration of Helsinki [42], and the EU General Data Protection Regulation (GDPR-EU 2016/679). All participants will provide written informed consent prior to their involvement, and personal information will be handled confidentially.

### 2.2. Study Setting

This study will take place in two distinct locations to ensure both controlled measurement conditions and practical application of the intervention. All pre- and post-intervention assessments will be conducted at the Laboratory of Neuromechanics, Department of Physical Education and Sport Science, Aristotle University of Thessaloniki in Serres, Greece. This laboratory is equipped with advanced measurement systems, including electromyography (EMG), ultrasound imaging, force plates, and biomechanical motion analysis tools, allowing for precise data collection under standardized conditions. Testing sessions will be scheduled at fixed intervals to maintain consistency in environmental factors and optimize the accuracy of results. The eight-week core training protocol will be carried out in two designated gyms in the city of Serres, both selected based on their suitability for structured strength and stability training. These gyms are specially equipped with basic tools such as resistance equipment, mats, exercise aids, and time-recording technology that facilitate the correct execution of exercises and the integrity of the intervention. All training sessions will be supervised by certified physical activity specialists who will ensure adherence to the study’s protocol and proper technical execution. The study is scheduled to begin in September 2025 and conclude in January or February 2026, following a structured timeline that ensures compliance with research design and minimizes external variability in training conditions. 

### 2.3. Eligibility Criteria

Participants for this study will be healthy, physically active adults between the ages of 25 and 35, engaging in regular recreational exercise or non-competitive sports activities. For the purpose of this study, the term “recreational exercise” was defined as physical activity engaged in a minimum of three times per week for a duration of at least one hour in each training session. A list of suitable activities is provided below, including but not limited to: aerobics, dance, folkloric dance, and swimming. A variety of outdoor activities and adventure sports are available, including orienteering, surfing, snowboarding, and caving. Racket games encompass a variety of sports, including tennis, paddle tennis, badminton, and squash. The participants were permitted to engage in a range of activities, including but not limited to mountain biking, mountain climbing, athletics, bowling, horseback riding, skateboarding, swimming, and hiking. Additionally, any other forms of exercise that the investigators deemed appropriate were permitted. In order to qualify for participation, an individual must have been continuously enrolled in the relevant programs for a minimum period of two years [41]. Inclusion is based on criteria ensuring suitability for core training interventions and reducing confounding variables related to prior structured stability training. Eligible participants must be in optimal health, demonstrating no recent musculoskeletal injuries or pre-existing conditions that could limit the full range of motion or prevent engagement in high-intensity exercise protocols. They must also commit to attending both baseline and final assessments and adhering to the structured eight-week intervention timeline to maintain data integrity.

Exclusion criteria include individuals diagnosed with musculoskeletal impairments that restrict functional movement or prevent core-specific engagement. Additionally, those with cardiovascular or metabolic conditions that contraindicate high-intensity exercise are excluded from participation. To maintain study validity, individuals already engaged in structured core training programs—such as Pilates, CrossFit, calisthenics, or athletes following systematic strength and stability regimens—will also be excluded to prevent prior training effects from influencing study outcomes.

### 2.4. Recruitment

Potential participants will be informed about the study through targeted outreach, including social media campaigns and physical posters placed in high-traffic locations such as gyms, sports facilities, and university premises. Individuals expressing interest will undergo an initial contact phase, where they will be provided with study information and invited to a one-on-one screening meeting with the research coordinator. This meeting will determine eligibility based on inclusion criteria, ensuring suitability for participation. Once eligibility is confirmed, participants will be thoroughly briefed on the preliminary and final screening procedures and asked to provide written informed consent for participation. However, the specific details of the eight-week core training protocol will not be disclosed at this stage to minimize potential bias. When the required number of participants is met, a computer-generated randomization process will allocate individuals into either the experimental group, which will undergo structured core training, or the control group, which will continue regular physical activity without targeted core interventions.

The experimental group will receive details of the training after randomization, and participants will need to sign additional written consent confirming their commitment to the intervention. The control group will receive written guidelines for a standardized two-day-per-week abs and back routine, and the program ensures controlled engagement without interfering with the core-specific intervention administered to the experimental group. Replacement participants will be recruited following the same screening and randomization procedures to maintain study integrity and adhere to predefined sample size calculations, in the event of participant withdrawal or noncompliance.

All participants will provide written informed consent prior to enrollment, and personal data will be handled in strict compliance with GDPR regulations to ensure confidentiality and participant protection (see Supplementary File S4).

**Figure 1:**
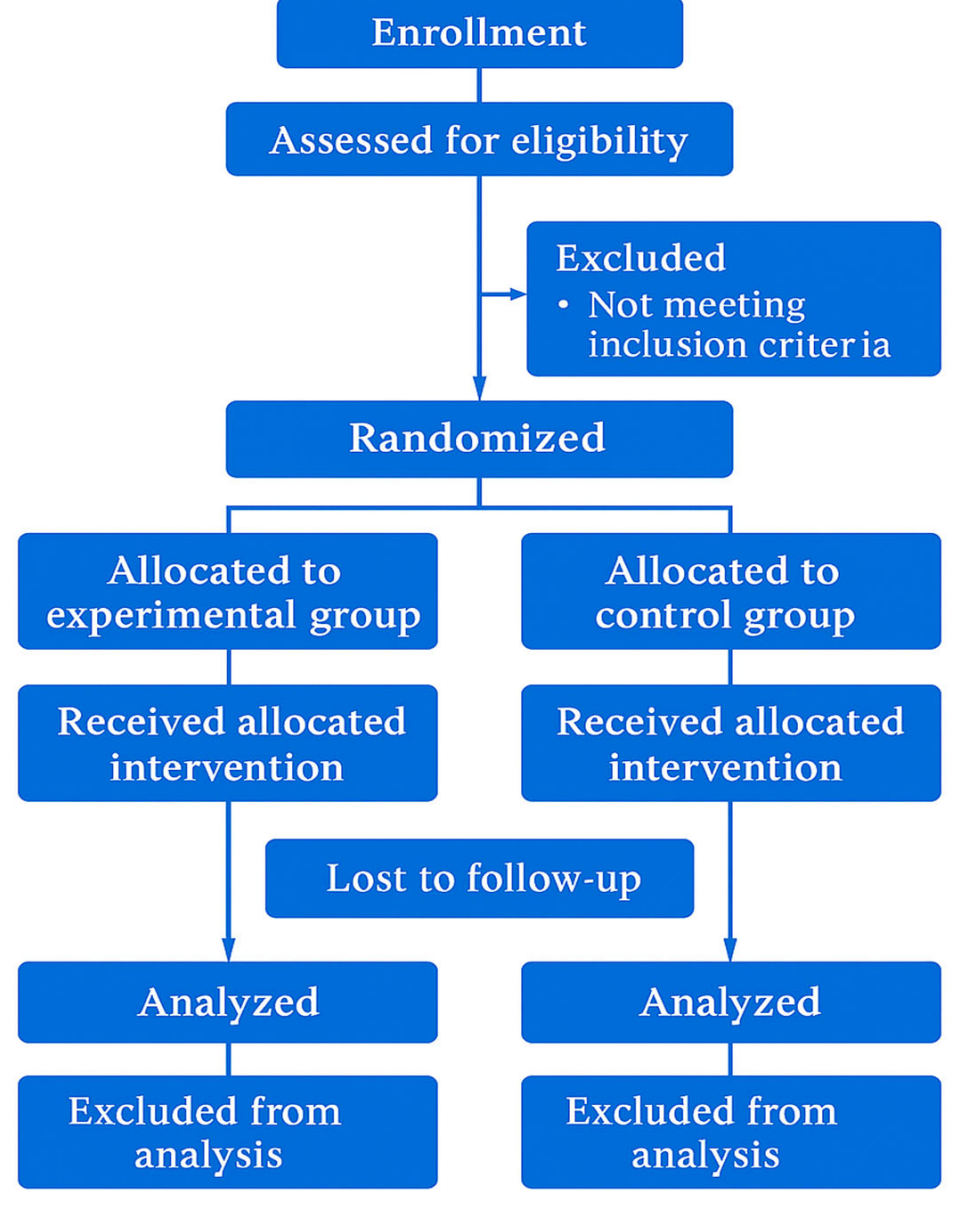
Hypothetical flowchart describing the planned participant progression throughout the study protocol. Designed in accordance with CONSORT and SPIRIT figure guidelines for protocol submissions.

A schematic representation of the planned participant trajectory (screening, eligibility, randomization, intervention, and follow-up) is illustrated in Figure 1. This flowchart is hypothetical and reflects the intended study structure prior to actual recruitment. No participant data is included at this stage, since enrollment is scheduled to begin in September 2025.

### 2.5. Sample Size Calculation

To determine the appropriate sample size for this study, a power analysis was conducted using G*Power 3.1 software [43]. The analysis was based on a repeated-measures design with two groups (experimental vs. control) measured at baseline and post-intervention. The following parameters were used: Effect Size (Partial η^2^) = 0.06 (medium effect); Cohen’s d = 0.50. Alpha Level (α) = 0.05 (5% significance threshold); power (1 − β) = 0.80 (80% probability of detecting a real effect); two measurement points (pre- and post-intervention); and two groups (experimental and control). According to the established criteria, the minimum requisite sample size for achieving adequate statistical power was computed to be 34 participants, with 17 participants allocated to each group. The objective of this study is to include and evaluate 40 participants, with 20 subjects in each group, to account for potential participant dropout. This methodological approach ensures that the study can detect meaningful differences in vertical ball strength, muscle activation (EMG), dynamic balance, angular velocity, muscle hypertrophy, and psychological impact (SWLS scores) with sufficient precision.

### 2.6. Description of the Study Instruments

This study employs a range of validated measurement instruments to accurately assess the effects of the eight-week core training intervention on muscle activation, hypertrophy, balance, and force application. To quantify the vertical impact force generated during the overhead medicine ball slam, a 3D dynamometer (Kistler, Winterthur, Switzerland) integrated with MARS performance analysis software will be used. This dynamometer will also be utilized to measure static balance parameters, ensuring a comprehensive analysis of lower limb stability under controlled conditions. EMG assessments will be conducted using a Biopac Systems Inc. machine (Goleta, CA, USA) with shielded surface electrode lead assemblies (model SS2, Biopac Systems Inc., Goleta, CA, USA) to record neuromuscular activity in the rectus abdominis (RA), external oblique abdominis (EO), and iliocostalis lumborum (IL) during exercise execution. EMG signals will be collected at a sampling rate of 1024 Hz using an amplifier/transmitter model TEL100 M (Biopak Systems Inc., Goleta, CA, USA), which exhibits a common mode rejection ratio exceeding 110 dB at 50/60 Hz, a bandwidth of 10–500 Hz, and an amplification gain of 1000. Data acquisition and processing will be performed using Acknowledge software (version 3.9.1, Biopac Systems) to ensure signal clarity and standardization across trials. Ultrasonography will be employed to measure deep core muscle thickness, particularly the transversus abdominis (TrA), internal oblique (IO), and lumbar multifidus (LM) at rest. These assessments will be performed using a computerized Aloka ProSound SSD 3500 SV ultrasound system (Aloka Co., Ltd., Tokyo, Japan) with a 6 cm transducer head operating at 13 MHz, ensuring high-resolution imaging of core musculature. Biomechanical analysis of torso and arm angular velocity during the medicine ball overhead slam will be conducted using Kinovea version 0.9.5 software (https://www.kinovea.org/download.html, accessed on 8 May 2025). This system will track angular momentum changes throughout the movement cycle, determining the final velocity of the ball at impact. High-definition motion capture will be facilitated using a PTZOptics Move 4K 20X NDI camera (PTZOptics, Downingtown, PA, USA) to ensure precision in kinematic assessments.

Anthropometric data, including height and body weight, will be recorded using an electronic balance with an integrated Seca stadiometer (Seca 220e, Hamburg, Germany). To evaluate the psychological impact of the intervention, participants will complete the Satisfaction with Life Scale (SWLS) questionnaire [44], providing insights into changes in subjective well-being throughout the study. The medicine balls used for overhead slam testing will be Energetics slam balls [(manufactured in Herzogenaurach, Germany, by Energetics (INTERSPORT Group))], ensuring consistent rebound and weight distribution across trials.

## 3. Procedure

### 3.1. Study Measuring Procedure

Both the experimental and control groups will undergo baseline and post-intervention measurements, scheduled at the neuromechanics laboratory of the Department of Physical Education and Sport Science, Aristotle University of Thessaloniki, in Serres. Testing sessions will be conducted by appointment, ensuring controlled environmental conditions across assessments. Upon arrival, participants’ anthropometric data—including weight, height, and age—will be recorded, alongside verification of personal contact details.

To optimize movement proficiency, participants will complete a brief warm-up routine in a designated laboratory space, ensuring sufficient joint mobility and neuromuscular readiness for the overhead medicine ball slam exercise. Following the warm-up, expert laboratory personnel will guide each subject through the measurement protocol, with a total session duration estimated between 30 and 40 min per participant.

The primary assessment will involve medicine ball overhead slam execution, during which force output will be recorded using a 3D dynamometer (Kistler, Winterthur, Switzerland) integrated with MARS performance analysis software. Simultaneously, EMG recordings will monitor muscle activation in the RA, EO, and IL, captured via bipolar surface electrodes (Biopac Systems Inc., Goleta, CA, USA). A high-definition PTZOptics Move 4K camera will document the angular velocity of the shoulder, hip, and knee joints from a standardized right-side view. To assess postural stability, participants will undergo a static balance evaluation, conducted using the Kistler 3D dynamometer while standing with eyes open. Additionally, deep-core muscle hypertrophy assessments will be performed via ultrasound imaging (Aloka ProSound SSD 3500 SV, Tokyo, Japan), measuring the TrA, IO, and LM at rest.

Psychological well-being will be evaluated using the Satisfaction with Life Scale (SWLS) questionnaire [45] to determine potential changes in the subjective quality of life resulting from the intervention.

### 3.2. Medicine Ball Overhead Slam Execution Protocol

Participants will follow precise movement instructions for Medicine Ball Overhead Slam to ensure consistency in execution.

CRITICAL STEPS:

1. Neutral Spine Alignment
  - Ensure the lumbar and thoracic spine remain neutral throughout the movement to prevent hyperextension or excessive flexion.
  - Engage the TrA and deep-core stabilizers prior to initiating the slam.
2. Controlled Upward Phase
  - Maintain a stacked ribcage position to prevent thoracic flare, ensuring optimal core bracing.
  - Avoid compensatory backward lean, ensuring spinal neutrality throughout the lift.
3. Explosive Downward Phase with Proper Bracing
  - Initiate forceful exhalation (bracing technique) before slamming the ball to stabilize intra-abdominal pressure and minimize lumbar compression.
  - Keep elbows slightly bent upon ball impact to dissipate kinetic force efficiently, protecting shoulder joints from excessive stress.
4. Ground Stability and Foot Positioning
  - Distribute weight evenly across the midfoot and heels to prevent forward displacement of force.
  - Maintain knee tracking aligned with toes, avoiding valgus collapse for joint integrity.
5. Follow-Through Mechanics
  - Ensure post-impact recovery is smooth, preventing uncontrolled movement deviations that may compromise kinetic efficiency.
  - Allow arms to fully extend post-slam, avoiding abrupt tension in the upper trapezius and neck stabilizers.

### 3.3. Validation and Muscle Activation in the Medicine Ball Overhead Slam

The overhead medicine ball slam is a validated measure of explosive upper-body power, core strength, and neuromuscular efficiency, making it a reliable test for our study parameters. Research has shown its strong correlation with upper-limb power output in swimmers [46], enhanced throwing velocity and precision in handball players [47], enhanced performance in rotational power sports [48], and increased explosive strength through ballistic training [49]. The Schultz Slam Test (SST) further supports its validity as a core power assessment in football [50], while studies in cricket [51] and golf [52] confirm its test-retest reliability for evaluating rotational power and movement efficiency. These findings collectively justify its use as a standardized performance metric in our study. The overhead medicine ball slam primarily activates core musculature, serving as the central force generator while stabilizing the lower body for efficient energy transfer through the upper limbs.

The RA, obliques, and TrA collectively facilitate spinal flexion, rotational control, and deep-core stabilization, ensuring efficient force transmission during the downward slam. The deltoids, triceps, and latissimus dorsi play a significant role in the overhead phase, contributing to arm extension and controlled force application. Lower-body stabilization relies on the quadriceps and gluteus maximus, which assist in squat positioning and upward propulsion, reinforcing postural control throughout the movement cycle. Additional secondary muscle groups contribute to balance and coordination, including the calves (gastrocnemius and soleus), rhomboids, and erector spinae. These muscles facilitate ankle stability, scapular positioning, and spinal alignment, preventing excessive compensatory movement and ensuring efficient kinetic chain integration.

This exercise relies on muscle synergy, where the core initiates power generation, directing force through the upper limbs, while the lower body establishes a stable base for execution. By optimizing intermuscular coordination, the medicine ball overhead slam enhances functional movement patterns, improving athletic explosiveness, postural integrity, and neuromuscular responsiveness.

### 3.4. Study Intervention Protocol

This core exercise protocol has been developed for implementation in health clubs or for recreational fitness practitioners who require a core workout to prevent back pain or to enhance their core strength. Detailed instructions for the exercises of the entire protocol (warm-up, main core training, and cool down) can be found in Supplementary File S1.

Important notice: The exercises can be varied or replaced by corresponding exercises depending on the level and needs of each person. It is important to maintain the sequence of exercises and the designated rest and activity time periods outlined in the protocol.

#### 3.4.1. Warm-Up Protocol for Core Training (Approx. 6 min)

The exercises selected for the warm-up phase were designed to prepare the muscular and central nervous systems to manage the specific loads demanded by the core training protocol. The exercises that comprise the warm-up are enumerated in Table 1.

#### 3.4.2. Main Core Training Protocol (Approx. 30–32 min)

The structured eight-week core training program (Table 2) follows an optimized sequence designed to activate, strengthen, and enhance dynamic movement efficiency, ensuring neuromuscular coordination and injury prevention. The protocol consists of eight exercise triads, each incorporating core stability, core strength, and high-intensity core power exercises, performed for 45 s per exercise (2.15 min per exercise triad), with progressively increasing rest periods between triads. The interval between the initial and secondary triad of exercises is one minute. The interval between the secondary and third triad of exercises is one minute and ten seconds, and it gradually increases to two minutes by the conclusion of the seventh triad of exercises (see Table 2). The progression of training within this protocol allows for individual adaptation based on the practitioner’s experience level. Novice practitioners may perform fewer triads or extend recovery periods to ensure proper neuromuscular adaptation and prevent excessive fatigue. This approach allows for a gradual increase in workload while maintaining movement integrity and control. In contrast, advanced practitioners can modify the protocol by increasing the total number of triads, reducing rest intervals, or integrating additional explosive movement variations to enhance power development and neuromuscular efficiency. Regardless of experience level, breathing techniques remain a crucial component of execution, with a focus on bracing and controlled exhalation. Proper breath control optimizes force transfer, reinforces spinal integrity, and minimizes compensatory movements, ensuring both safety and effectiveness throughout the training process.

The structured progression of core training follows established principles to optimize neuromuscular adaptation. Beginning with core stability exercises, deep core muscles are activated to improve postural control and spinal alignment. Research has shown that stabilizing musculature plays a crucial role in force transmission and movement efficiency, emphasizing the importance of initiating training with stability-focused movements [2]. This phase prepares the kinetic chain for increased mechanical demand, reducing compensatory movements. These movements are executed at a controlled aerobic pace, emphasizing bracing and deep core engagement rather than rapid force production.

Following stability work, core strength exercises enhance force production and neuromuscular coordination, benefiting from pre-activated stabilizers. Strength training contributes to movement efficiency and biomechanical stability, supporting the transition from foundational activation to controlled force output [4]. This progression allows for improved kinetic chain integration, reinforcing motor control. These exercises follow a standard tempo, incorporating core bracing techniques, lateral breathing mechanics, and controlled execution to maximize muscle recruitment and stability.

The final phase of the sequence consists of high-intensity core power exercises, designed to maximize explosive force output and agility. Research on force–velocity relationships confirms that progressive sequencing from stability to strength to power enhances neuromuscular efficiency and reactive performance [3]. By concluding with high-intensity movement, the protocol ensures optimized power application, reinforcing dynamic stability and responsiveness. These exercises are performed above the anaerobic threshold, utilizing fast-paced movements with high muscular recruitment to promote optimal neuromuscular adaptation.

The selection of 45 s per exercise is based on scientific evidence supporting short-duration high-intensity training, which optimizes neuromuscular activation, endurance, and power development. Studies indicate that exercise bouts lasting between 30 and 60 s enhance muscle engagement and metabolic efficiency, making this duration ideal for core stability, strength, and power exercises [53]. Additionally, the total triad duration of 2 min and 15 s balances intensity and recovery, ensuring optimal neuromuscular engagement while preventing excessive fatigue, aligning with structured progression principles [54].

#### 3.4.3. Stretching and Cool-Down Protocol (Approx. 5 min)

Table 3 presents an overview of the exercises designed to enhance and preserve the flexibility of muscles targeted by the primary segment of the protocol.

#### 3.4.4. Control Group Core Exercise Routine

To maintain participant engagement while ensuring minimal external variability, the control group will follow a standardized two-day-per-week abs and back exercise routine. This program does not replicate the experimental group’s structured core training but provides foundational neuromuscular activation for controlled comparisons. The routine consists of warm-up, primary exercises, and cool-down phases totaling 20–25 min per session. Specific exercise descriptions can be found in Supplementary File S1.

To guarantee minimal neuromuscular adaptation while sustaining engagement and adherence, the control group will adhere to a standardized abs and back exercise routine performed twice per week, with each session lasting approximately 20–25 min. The program encompasses a range of exercises with moderate to low intensity, focusing on the anterior and posterior trunk musculature (e.g., basic crunches, seated back extensions, static planks). However, it does not include structured progression or explosive core activation tasks. This approach intentionally avoids multi-planar, high-intensity movements, thereby allowing the protocol to function as a minimal-stimulus comparator. The objective of this study is to differentiate targeted core-specific neuromuscular adaptations elicited by the experimental protocol from general movement-based engagement. The intensity and frequency parameters were designed to ensure that the core musculature remains active at a basic functional level, without triggering measurable changes in hypertrophy, balance, or dynamic power capacity.

This controlled baseline routine has been demonstrated to enhance the interpretability of group comparisons and support internal validity by reducing confounding variability associated with total training volume or unintended adaptive responses.

### 3.5. Statistical Analysis

All statistical analyses will be conducted using IBM SPSS Statistics (version 29, IBM Corp., Armonk, NY, USA). Normality checks will be performed via the Shapiro–Wilk test (small to medium samples) supplemented by histograms, Q-Q plots, and skewness/kurtosis assessments.

Reliability Analysis:

- SWLS questionnaire consistency will be assessed using Cronbach’s alpha (>0.70).
- Item-total correlations will ensure meaningful contributions to overall scoring.

Inferential Testing:

- Within-group comparisons: paired samples *t*-test (normal data) or Wilcoxon Signed-Rank test (non-parametric).
- Between-group comparisons: independent samples *t*-test (parametric) or Mann–Whitney U test (non-parametric).
- Repeated-measures ANOVA will assess pre- and post-intervention changes, applying Bonferroni corrections for multiple comparisons.

Effect Size and Correlations:

- Cohen’s d for *t*-tests, Partial Eta Squared (η^2^) for ANOVA.
- Pearson’s correlation (parametric) or Spearman’s rank correlation (non-parametric) to explore variable associations.

Additional Measures:

- Homogeneity of variance was tested via Levene’s test.
- Intention-to-treat (ITT) analysis for participant dropout management, using multiple imputation techniques to mitigate bias.

## 4. Expected Results

The intervention is anticipated to yield improvements in core muscle activation, particularly in the transverse abdominis, internal oblique, and lumbar multifidus, contributing to spinal resilience and segmental control. Static balance is expected to improve via enhanced neuromuscular coordination and recruitment efficiency, while higher force outputs are projected during medicine ball slam trials, indicating improved motor control and kinetic chain engagement. Beyond musculoskeletal outcomes, the intervention is expected to elicit beneficial neural and metabolic adaptations. These may include enhanced intermuscular coordination, improved regulation of joint angular velocity, and increased corticospinal excitability—factors that collectively contribute to more efficient motor unit recruitment and refined movement control. Furthermore, the application of core bracing techniques may facilitate intra-abdominal pressure modulation, enhance spinal stability, and lead to improved task-specific energy efficiency during dynamic full-body movements. These neuromechanical enhancements are anticipated to play a central role in the observed performance and balance improvements.

### 4.1. Neuromuscular and Biomechanical Adaptations

The structured eight-week core training program is expected to elicit significant neuromuscular improvements, enhancing force application, muscle activation, and movement efficiency. These balance and stability adaptations are particularly relevant to public health, as they contribute to fall prevention, improved daily functional mobility, and reduced risk of musculoskeletal disorders, particularly in populations at risk for postural deficits and movement imbalances.

- Force Output Enhancement: The experimental group is anticipated to demonstrate increased vertical ball strength during the medicine ball overhead slam, reflecting enhanced trunk power and kinetic force transfer. This expectation aligns with findings from Saeterbakken et al. [24], who reported improvements in throwing velocity and explosive power following dynamic core training.
- Muscle Activation and Neural Efficiency: EMG analysis is expected to reveal greater activation of the RA, EO, and IL during explosive movements. Studies suggest that core-specific neuromuscular training enhances motor unit recruitment, optimizing stabilization and force transfer mechanisms [23].
- Kinetic Chain Coordination: Previous research indicates that multi-joint movements elicit higher neuromuscular engagement compared to isolated core exercises, reinforcing the hypothesis that medicine ball slams will enhance intermuscular synchronization [29].

These adaptations will collectively contribute to functional performance improvements, reinforcing core training integration within sports-specific and recreational movement patterns. This aligns with previous research highlighting the importance of functional kinetic chain activation for optimizing athletic performance [22,26].

### 4.2. Balance and Stability Enhancements

The structured core training intervention is expected to produce measurable improvements in postural stability, proprioception, and neuromuscular control, reinforcing functional movement efficiency.

- Postural Control and Static Balance: Participants are anticipated to exhibit enhanced static balance, demonstrated by greater center-of-pressure stability in force plate assessments. Research has shown that progressive core stabilization drills improve proprioceptive feedback, reducing postural sway [27].
- Kinesthetic Awareness and Motor Coordination: The intervention is expected to refine sensorimotor integration, optimizing kinesthetic awareness and neuromuscular responsiveness during high-intensity dynamic movements. Studies confirm that core-focused neuromuscular training improves reaction time and stability in functional tasks [28].
- Dynamic Balance Adaptations: Structured multi-plane core exercises are likely to yield greater dynamic stability improvements, surpassing traditional static-only interventions. Previous findings indicate that dynamic balance testing is more sensitive in detecting neuromuscular adaptations compared to static assessments [34].

Furthermore, evidence suggests that dynamic balance assessments are more sensitive in detecting training-induced adaptations compared to static evaluations, reinforcing the expectation that participants will exhibit measurable improvements in force plate stability tests following the intervention [26].

### 4.3. Biomechanical Changes in Movement Efficiency

The present study hypothesizes that the eight-week core training protocol will enhance torso and arm angular velocity. This, in turn, is expected to improve kinetic chain coordination, power transmission, and movement accuracy in medicine ball slam executions.

- Angular Velocity Optimization: The experimental group is anticipated to exhibit increased peak angular velocities in shoulder, hip, and knee joints, reflecting greater rotational force production and coordinated energy transfer. Previous studies indicate that higher joint angular velocities contribute to explosive athletic performance, particularly in ballistic movements like overhead throws and vertical jumps [55].
- Kinetic Chain Integration: Biomechanical tracking through Kinovea software is expected to confirm refined movement sequences, showing improved power transmission efficiency from core activation to upper-limb execution. Research has demonstrated that joint moments and angular velocity interplay significantly impact trunk acceleration dynamics, reinforcing the role of core training in movement efficiency [56,57].
- Neuromuscular Responsiveness: The intervention is expected to enhance intermuscular synchronization, optimizing joint stability and movement control during ballistic core-driven exercises. Studies highlight that structured neuromuscular training improves peak velocity control, leading to greater movement precision and optimized force application [57].

These biomechanical enhancements will collectively reinforce explosive movement mechanics, supporting athletic performance applications and general fitness adaptations.

### 4.4. Muscle Hypertrophy and Structural Improvements

It is anticipated that the eight-week core training protocol will elicit structural adaptations, including enhanced deep-core muscle thickness and lumbar stability, as measured through ultrasound imaging. Such structural improvements hold significant public health value, supporting injury prevention, spinal longevity, and movement resilience across recreational, occupational, and aging populations. Progressive core training could contribute to reducing the prevalence of lower back pain and musculoskeletal dysfunctions, which are leading causes of work-related disabilities.

- Deep-Core Muscle Hypertrophy: The intervention is anticipated to yield measurable increases in the thickness of the TrA, IO, and LM. Prior research confirms that structured core training improves TrA thickness, reinforcing spinal support and postural endurance [30].
- Spinal Stability and Postural Integrity: Progressive loading combined with bracing and hollowing techniques is expected to optimize core muscle activation, minimizing lower back dysfunction and enhancing long-term lumbar resilience. Studies indicate that lumbar stabilization training significantly improves muscle recruitment patterns, contributing to postural control and injury prevention [58].
- Ultrasound Validation of Core Adaptations: Real-time ultrasound imaging (RUSI) will confirm training-induced hypertrophy, supporting evidence that deep-core muscle activation enhances spinal mechanics and functional movement efficiency [58].

Collectively, these adaptations will enhance neuromuscular control, augment postural endurance, and fortify core structural stability. Consequently, this will have a beneficial impact on both athletic performance and preventive health strategies.

### 4.5. Psychological and Functional Outcomes

The eight-week core training program is hypothesized to engender positive psychological adaptations, including enhanced self-efficacy, movement confidence, and overall life satisfaction among program participants. These psychological benefits extend beyond individual training effects, contributing to public health initiatives aimed at improving mental resilience, stress reduction, and overall movement confidence. Structured exercise programs have been shown to enhance mental well-being and social engagement, reinforcing their role in health promotion strategies.

- Subjective Well-Being and Motivation: The Satisfaction with Life Scale (SWLS) questionnaire is expected to show improvements in perceived quality of life, reinforcing the link between structured exercise engagement and mental well-being. Studies have demonstrated that regular physical activity positively influences life satisfaction, particularly in individuals adhering to progressive training models [59].
- Self-Efficacy and Training Adherence: The structured progression of the core training protocol is likely to foster higher self-efficacy, promoting greater motivation and engagement in physically demanding tasks. Research confirms that exercise adherence improves when participants perceive tangible benefits in movement efficiency and physical resilience [60].
- Neuromuscular Confidence and Movement Optimization: As postural control and core stability improve, participants are expected to experience greater movement confidence, reinforcing long-term training sustainability. Previous findings indicate that enhanced neuromuscular control contributes to reduced movement anxiety, leading to greater exercise enjoyment and continued participation [61].

These psychological and functional adaptations will collectively support participant well-being, reinforcing long-term health benefits beyond neuromuscular training effects.

### 4.6. Comparative Analysis and Study Implications

The structured eight-week core training protocol is expected to yield substantial post-intervention differences between the experimental and control groups, thereby reinforcing the effectiveness of targeted core interventions.

- Neuromuscular and Biomechanical Adaptations: The experimental group is anticipated to display greater muscle activation, force output, and core-driven movement efficiency, surpassing baseline measures and outperforming the control group in medicine ball slam performance and EMG activation profiles [24].
- Balance and Postural Control Enhancements: Quantifiable improvements in static and dynamic balance tests are expected in trained individuals, demonstrating superior lower-limb stabilization and kinetic chain coordination compared to non-core-trained participants [28].
- Structural and Psychological Benefits: Increased deep-core muscle thickness, spinal endurance, and subjective life satisfaction are projected for trained participants, reinforcing both physical resilience and mental well-being through structured core activation strategies [30,61].

These findings will support the scalability of core training methodologies beyond elite athletic populations, providing valuable applications for recreational fitness, rehabilitation, and preventive health strategies [62,63].

## 5. Study Limitations

There are potential limitations that should be acknowledged to strengthen transparency and validity. Here are some key limitations to consider:

- Sample Size Constraints: Despite the power analysis confirming the minimum sample size of 34 participants, this relatively small cohort may limit the detection of subtle training effects, reducing generalizability to larger, diverse populations.
- While the inclusion of healthy adults aged 25–35 provides a physiologically stable cohort for detecting training-specific adaptations, it also limits the generalizability of findings to other age groups. This age bracket was deliberately chosen to mitigate the impact of maturation-related neuromuscular developments, which could otherwise compromise the interpretation of results. Subsequent studies should endeavor to extend the age range in order to investigate the protocol’s influence across a more extensive range of demographics, including older adults and sedentary populations.
- Intervention Duration: An eight-week timeframe may be sufficient to capture short-term neuromuscular and biomechanical adaptations, but it may not fully reflect long-term hypertrophic changes, particularly in deep-core muscle development
- Participant Adherence and Training Variability: Differences in individual recovery rates, motivation, and external physical activity levels may introduce inconsistencies in training adherence, affecting final outcome measures.
- Measurement Variability: While EMG, force plate, ultrasound imaging, and motion tracking provide objective data, inherent technical limitations (such as electrode placement sensitivity, force plate calibration, and operator precision in ultrasound scans) may introduce minor variability in recorded results.
- Control Group Considerations: Participants in the control group will not undergo structured core training, but external exercise habits beyond the study’s scope may influence baseline and post-intervention assessments, creating additional variability.
- Lack of Long-Term Follow-Up: While pre- and post-intervention assessments provide insights into immediate improvements, the absence of extended post-study evaluations limits our ability to determine the retention of training adaptations over months or years.
- It is acknowledged that the absence of pilot data constitutes a significant limitation. The designated laboratory was already reserved for other scheduled research activities prior to the commencement of the trial period, which precluded the execution of a pilot phase. A comprehensive preparatory pilot study would have necessitated an estimated four months of recruitment, analysis, and evaluation time.

Recognizing these limitations ensures transparency in findings, guiding future research toward long-term investigations, larger sample sizes, and refined measurement methodologies.

## 6. Practical Implications

The structured core training protocol in this study is designed to enhance spinal stability, core strength, and dynamic movement efficiency, making it highly applicable across diverse fitness, rehabilitation, and athletic performance settings. Its implementation extends beyond general fitness, offering tangible benefits in injury prevention, postural correction, and neuromuscular optimization. Additionally, structured core training plays a crucial role in public health initiatives, contributing to injury prevention, improved functional movement, and musculoskeletal health. By addressing core weaknesses and postural imbalances, such interventions help reduce the long-term burden of sedentary-related dysfunctions in both clinical and occupational settings. In health clubs and recreational fitness, this protocol can be integrated into group training programs, providing a progressive core training system tailored for individuals seeking to enhance core function and reduce injury risk. Personal trainers can adapt the exercises into individualized plans, addressing core weaknesses, posture imbalances, and lower back pain prevention.

Athletes in rotational and explosive sports—such as tennis, golf, martial arts, and track and field—can leverage this protocol to optimize kinesthetic awareness, neuromuscular coordination, and peak force generation. The high-intensity core power exercises, performed at higher effort thresholds, enhance reactivity, coordination, and directional change ability, supporting disciplines reliant on agility-based movements.

Beyond fitness applications, the protocol has significant rehabilitation potential, particularly for individuals recovering from lower back injuries or postural imbalances. This rehabilitation-focused approach aligns with public health strategies, offering structured movement interventions to reduce lower back dysfunction, improve spinal integrity, and enhance mobility across diverse populations. Such preventive methodologies are increasingly recognized as essential tools in reducing healthcare costs associated with musculoskeletal disorders. Its structured progression of stability, strength, and high-intensity exercises helps mitigate re-injury risk while reinforcing lumbar control and neuromuscular endurance. Exercises such as planks, bird dog drills, and lateral step jumps can be seamlessly integrated into physical therapy regimens, promoting spinal integrity and functional movement efficiency.

Given the prevalence of postural strain in sedentary workplaces, this protocol may serve as a preventative tool for office workers experiencing musculoskeletal discomfort due to prolonged sitting. Workplace-related musculoskeletal disorders are a growing public health concern, with prolonged sedentary behavior linked to postural dysfunction and chronic lower back pain. Integrating core stability and strength exercises into workplace wellness programs can offer preventive solutions, reducing strain-related injuries and improving long-term spinal health. Integrating targeted core stability and strength exercises can enhance postural endurance, reducing strain-related injuries and improving functional resilience.

The protocol also holds academic and research significance, serving as a foundation for studying neuromuscular adaptations in exercise science programs. The incorporation of EMG assessments, force plate data, and angular velocity tracking strengthens its applicability in biomechanical research, facilitating further exploration of core activation mechanisms, movement efficiency, and functional adaptations. In summary, this structured core training protocol aims to offer a scalable, evidence-based framework for enhancing neuromuscular function, postural control, and perceived well-being in recreationally active adults, with potential applications extending beyond fitness to broader public health domains.

## 7. Conclusions

This protocol outlines a structured, evidence-informed core training intervention designed to enhance neuromuscular activation, movement control, deep-core hypertrophy, and perceived well-being among recreationally active adults. Grounded in principles of progressive motor complexity and functional integration, the proposed framework seeks to bridge the gap between high-performance athletic methodologies and scalable public health applications. If supported by future data, this approach may offer a replicable and adaptable model for improving injury prevention, musculoskeletal resilience, and long-term functional quality of life across diverse non-athlete populations.

## Figures and Tables

**Table 1:** Warm-up protocol for core training (approx. 6 min).

Exercise Name	Distance/Duration	Repetitions
Suitcase Walk (forward/backward)	15 m + 15 m	4 reps
Overhead Dumbbells Walk	15 m + 15 m	2 reps
Walking 5 m + High Knee Jog	5 m + 10 m	4 reps
Bend Over T-Rotation	30 s	FT
Squat Lateral Reach + Squat Side Reach	45 s	FT
Lying Bend Leg Twist	30 s	FT
Bridge Exercise	30 s	FT
Knee Push-ups	30 s	FT

reps = repetitions, s = seconds, m = meters, FT = for the specific time with self-regulated intensity, approx. = approximately. The instructions for the exercises can be found in Supplemental File S1.

**Table 2:** Main Core Training Protocol Structured into Exercise Triads (Approx. 30–32 min).

Exercise Triad	Core Stability Exercises	Core Strength Exercises	High-Intensity Core Power Exercises	Rest Period
1st Triad	Squat + Cross Knees to Elbows	Bird Dog Exercise	Three Repeat Ice Skaters + One High Jump	1:00 min
2nd Triad	Standing to Walkout Plank	Dead Bug Exercise	Side to Side Lateral Floor Tap (3 m)	1:10 min
3rd Triad	Standing Dumbbell Windmill	Sit-ups	Lateral Step Jump with Up-Down Exercise	1:20 min
4th Triad	Side to Side Beast Crawl	Reverse Crunch Abs	Jack Squat + Twist Jump	1:30 min
5th Triad	Front Lunge Body Twist	Alternative Side Plank	Running 3 m Forward and Backward with Floor Tap	1:40 min
6th Triad	Downward Dog to Cobra	Superman Exercise with Arm and Leg Lateral Extension	Lateral 3 Steps High Knees Shuffle	1:50 min
7th Triad	Prisoner Get-Up Exercise	Front Plank Moving Dumbbell Left–Right	Burpee Broad Jump	2:00 min
8th Triad	Thruster with Dumbbell	Russian Twist with Dumbbell	Explosive Jumping Alternating Lunges	Stretching

min = minute. Approx = approximately, Triad = structured group of three consecutive core exercises (stability, strength, power). The instructions for the exercises can be found in Supplemental File S1. Each exercise lasts 45 s. A group of three exercises lasts 2 min and 15 s.

**Table 3:** Stretching and cool-down protocol (approx. 5 min).

Exercise Name	Duration
Standing Rolling Down Stretch	20 s
Hip Flexor Stretches (alternating sides)	20 s each side
Cat Camel Stretches	20 s
Threading the Needle Back Stretch	40 s
Child’s Pose Stretches	20 s
Scorpion Stretch (alternating sides)	20 s each side
Cobra Extension Stretch	20 s

s = second. The instructions for the exercises can be found in Supplemental File S1.

## Data Availability

The data presented in this study are available upon request from the corresponding author, due to ethical and privacy reasons.

## Supplementary Materials

The following supporting information can be downloaded at https://www.mdpi.com/article/10.3390/mps8040077/s1, Supplementary File S1: Protocol exercises for both groups; Supplementary File S2: ClinicalTrials.gov Registration, SPIRIT, CERT, TIDiER; Supplementary File S3: GDPR & Consent Form Details; Supplementary File S4: Ethics Committee Approval.

## Abbreviations

The following abbreviations are used in this manuscript:

EMG	Electromyography
Reps	Repetitions
Sec	Seconds
FT	For the specific time with self-regulated intensity
Approx.	Approximately
SWLS	Satisfaction With Life Scale
