# Supplementary material for: Development and Implementation of a Core Training Protocol: Effects on Muscle Activation, Hypertrophy, Balance, and Quality of Life in Recreationally Active Adults
Source: Methods Protoc. 2025 Jul 8;8(4):77. doi: 10.3390/mps8040077 (PMC12286126; doi:10.3390/mps8040077)
Supplement: Supplementary file 1 [file mps-08-00077-s001.zip › Supplemetary files/mps-3708003 Clinicaltrials.gov SPIRIT CERT TIDiER supplementary 2.pdf]

- https://clinicaltrials.gov/ct2/show/NCT07025395,

ClinicalTrials.gov

Clinical Trial Registration Statement

The present study, titled *CORE Training for Mus*culoskeletal Health: A Study on Adaptive Exercise Protocols*, has been prospectively registered on *ClinicalTrials.gov* with the foldling details:

Brief Title: CORE Training for Musculoskeletal Health: A Study on Adaptive Exercise Protocols

Registration ID (NCT Number): NCT07025395

Unique Protocol ID: AUTH-CORE-STUDY

Principal Investigator / Record Owner: Ioannis Tsartsakis

Initial Release Date: June 10, 2025

Last Updated: June 19, 2025

Record Status: Released to Public

The study has been classified as a non-ACT (non–FDA-regulated intervention). The complete registration file, including protocol components and study-specific metadata, is publicly accessible via the ClinicalTrials.gov public record.

A PDF copy of the full trial registration record, as expoted from [illegible]

STANDARD PROTOCOL ITEMS: RECOMMENDATIONS FOR INTERVENTIONAL TRIALS

SPIRIT 2013 Checklist: Recommended items to address in a clinical trial protocol and related documents*

Section/item	ItemNo	Description	Location in Manuscript
**Administrative information**			
Title	1	Descriptive title identifying the study design, population, interventions, and, if applicable, trial acronym	Title Page
Trial registration	2a	Trial identifier and registry name. If not yet registered, name of intended registry	2.1 Trial Design
	2b	All items from the World Health Organization Trial Registration Data Set	N/A
Protocol version	3	Date and version identifier	2.1 Trial Design
Funding	4	Sources and types of financial, material, and other support	Funding: This research received no external funding.
Roles and responsibilities	5a	Names, affiliations, and roles of protocol contributors	Author Section
	5b	Name and contact information for the trial sponsor	N/A
	5c	Role of study sponsor and funders, if any, in study design; collection, management, analysis, and interpretation of data; writing of the report; and the decision to submit the report for publication, including whether they will have ultimate authority over any of these activities	N/A

	5d	Composition, roles, and responsibilities of the coordinating centre, steering committee, endpoint adjudication committee, data management team, and other individuals or groups overseeing the trial, if applicable (see Item 21a for data monitoring committee)	N/A
**Introduction**			
Background and rationale	6a	Description of research question and justification for undertaking the trial, including summary of relevant studies (published and unpublished) examining benefits and harms for each intervention	1. Introduction
	6b	Explanation for choice of comparators	1. Introduction
Objectives	7	Specific objectives or hypotheses	1. Introduction
Trial design	8	Description of trial design including type of trial (eg, parallel group, crossover, factorial, single group), allocation ratio, and framework (eg, superiority, equivalence, noninferiority, exploratory)	2.1 Trial Design
**Methods: Participants, interventions, and outcomes**			
Study setting	9	Description of study settings (eg, community clinic, academic hospital) and list of countries where data will be collected. Reference to where list of study sites can be obtained	2.2 Study Setting

Eligibility criteria	10	Inclusion and exclusion criteria for participants. If applicable, eligibility criteria for study centres and individuals who will perform the interventions (eg, surgeons, psychotherapists)	2.3 Eligibility Criteria
Interventions	11a	Interventions for each group with sufficient detail to allow replication, including how and when they will be administered	3.3 Study Intervention Protocol
	11b	Criteria for discontinuing or modifying allocated interventions for a given trial participant (eg, drug dose change in response to harms, participant request, or improving/worsening disease)	N/A
	11c	Strategies to improve adherence to intervention protocols, and any procedures for monitoring adherence (eg, drug tablet return, laboratory tests)	N/A
	11d	Relevant concomitant care and interventions that are permitted or prohibited during the trial	N/A
Outcomes	12	Primary, secondary, and other outcomes, including the specific measurement variable (eg, systolic blood pressure), analysis metric (eg, change from baseline, final value, time to event), method of aggregation (eg, median, proportion), and time point for each outcome. Explanation of the clinical relevance of chosen efficacy and harm outcomes is strongly recommended	3.3 Study Intervention Protocol

Participant timeline	13	Time schedule of enrolment, interventions (including any run-ins and washouts), assessments, and visits for participants. A schematic diagram is highly recommended (see Figure)	3.3 Study Intervention Protocol
Sample size	14	Estimated number of participants needed to achieve study objectives and how it was determined, including clinical and statistical assumptions supporting any sample size calculations	2.5 Sample Size Calculation
Recruitment	15	Strategies for achieving adequate participant enrolment to reach target sample size	2.4 Recruitment
**Methods: Assignment of interventions (for controlled trials)**			
Allocation:			
Sequence generation	16a	Method of generating the allocation sequence (eg, computer-generated random numbers), and list of any factors for stratification. To reduce predictability of a random sequence, details of any planned restriction (eg, blocking) should be provided in a separate document that is unavailable to those who enrol participants or assign interventions	3.1 Study Measuring Procedure
Allocation concealment mechanism	16b	Mechanism of implementing the allocation sequence (eg, central telephone; sequentially numbered, opaque, sealed envelopes), describing any steps to conceal the sequence until interventions are assigned	3.1 Study Measuring Procedure

Implementation	16c	Who will generate the allocation sequence, who will enrol participants, and who will assign participants to interventions	N/A
Blinding (masking)	17a	Who will be blinded after assignment to interventions (eg, trial participants, care providers, outcome assessors, data analysts), and how	N/A
**Methods: Data collection, management, and analysis**			
Data collection methods	18a	Plans for assessment and collection of outcome, baseline, and other trial data, including any related processes to promote data quality (eg, duplicate measurements, training of assessors) and a description of study instruments (eg, questionnaires, laboratory tests) along with their reliability and validity, if known. Reference to where data collection forms can be found, if not in the protocol	2.6 Description of Study Instruments
	18b	Plans to promote participant retention and complete follow-up, including list of any outcome data to be collected for participants who discontinue or deviate from intervention protocols	3.3 Validation and Muscle Activation
Data management	19	Plans for data entry, coding, security, and storage, including any related processes to promote data quality (eg, double data entry; range checks for data values). Reference to where details of data management procedures can be found, if not in the protocol	2.6 Description of Study Instruments
Statistical methods	20a	Statistical methods for analysing primary and secondary outcomes. Reference to where other details of the statistical analysis plan can be found, if not in the protocol	3.4 Statistical Analysis

	20b	Methods for any additional analyses (eg, subgroup and adjusted analyses)	3.4 Statistical Analysis
**Methods: Monitoring**			
Data monitoring	21	Composition of data monitoring committee (DMC); summary of its role and reporting structure; statement of whether it is independent from the sponsor and competing interests; and reference to where further details about its charter can be found, if not in the protocol. Alternatively, an explanation of why a DMC is not needed	N/A
**Ethics and dissemination**			
Research ethics approval	24	Plans for seeking research ethics committee/institutional review board (REC/IRB) approval	Under Licence approval
Protocol amendments	25	Plans for communicating important protocol modifications (eg, changes to eligibility criteria, outcomes, analyses) to relevant parties (eg, investigators, REC/IRBs, trial participants, trial registries, journals, regulators)	5. Study Limitations
Consent or assent	26a	Who will obtain informed consent or assent from potential trial participants or authorised surrogates, and how (see Item 32)	2.4. Recruitment
	26b	Additional consent provisions for collection and use of participant data and biological specimens in ancillary studies, if applicable	N/A

Confidentiality	27	How personal information about potential and enrolled participants will be collected, shared, and maintained in order to protect confidentiality before, during, and after the trial	2.3. Eligibility Criteria
Declaration of interests	28	Financial and other competing interests for principal investigators for the overall trial and each study site	Ethics Statement
Access to data	29	Statement of who will have access to the final trial dataset, and disclosure of contractual agreements that limit such access for investigators	Ethics Statement
Ancillary and post-trial care	30	Provisions, if any, for ancillary and post-trial care, and for compensation to those who suffer harm from trial participation	N/A
Dissemination policy	31a	Plans for investigators and sponsor to communicate trial results to participants, healthcare professionals, the public, and other relevant groups (eg, via publication, reporting in results databases, or other data sharing arrangements), including any publication restrictions	4.6 Comparative Analysis & Study
	31b	Authorship eligibility guidelines and any intended use of professional writers	N/A
	31c	Plans, if any, for granting public access to the full protocol, participant-level dataset, and statistical code	N/A
**Appendices**			
Informed consent materials	32	Model consent form and other related documentation given to participants and authorised surrogates	Supplementary File 2

Biological specimens	33	Plans for collection, laboratory evaluation, and storage of biological specimens for genetic or molecular analysis in the current trial and for future use in ancillary studies, if applicable	N/A

*It is strongly recommended that this checklist be read in conjunction with the SPIRIT 2013 Explanation & Elaboration for important clarification on the items. Amendments to the protocol should be tracked and dated. The SPIRIT checklist is copyrighted by the SPIRIT Group under the Creative Commons "Attribution-NonCommercial-NoDerivs 3.0 Unported" license.

CERT ✓ Consensus on Exercise Reporting Template

A Checklist for what to include when reporting exercise programs

Section / Topic	Item	Checklist item	Location ** Primary paper (page or appendix number)	† Other (paper or protocol, website (URL)
**WHAT: materials**	**1.**	Detailed description of the type of exercise equipment (e.g. weights, exercise equipment such as machines, treadmill, bicycle ergometer etc<br>✓ Medicine balls (used for power-based exercises)<br>✓ Balance boards (for proprioception training)<br>✓ Resistance bands (for core stabilization drills)<br>✓ Dynamometers (Kistler 3D) (to measure force output)<br>✓ Electromyography (EMG) devices (for muscle activation monitoring)<br>✓ Ultrasound imaging (for assessing core muscle hypertrophy)	2.6. Description of the Study Instruments, & 3.3 Study Intervention Protocol	
**WHO: provider**	**2.**	Detailed description of the qualifications, teaching/supervising expertise, and/or training undertaken by the exercise instructor<br>✓ Lead researcher: Ioannis Tsartsapakis (Exercise Science Specialist)<br>✓ Biomechanics & EMG Technicians: Trained professionals in neuromuscular assessments<br>✓ Strength & Conditioning Coaches: Certified experts in core training methods<br>This multidisciplinary team ensures proper execution, monitoring, and methodological integrity throughout the study."_	Study Settings (2.2)	
**HOW: delivery**	**3.**	Describe whether exercises are performed individually or in a group	Study Settings (2.2)	

	✓ Exercises are performed in small-group settings<br>✓ All sessions are fully supervised by trained professionals<br>✓ Real-time feedback provided through EMG and ultrasound assessments		
**4**.	Describe whether exercises are supervised or unsupervised and how they are delivered<br>✓ Fully supervised sessions led by certified physical activity specialists, including the lead researcher and biomechanics experts.<br>✓ Small-group settings allow for real-time individualized feedback and technique correction.<br>✓ EMG and ultrasound imaging assessments provide detailed neuromuscular activation tracking.	Methods Section → Study Settings (2.2)	
**5.**	Detailed description of how adherence to exercise is measured and reported<br>✓ Session attendance logs will track participant consistency.<br>✓ Performance monitoring through biomechanical and neuromuscular feedback mechanisms (force plate, EMG, ultrasound).<br>✓ Participant compliance evaluated weekly, ensuring adherence to intervention guidelines.	Methods Section→ Study Settings (2.2)	
**6.**	Detailed description of motivation strategies<br>✓ Structured progression model encourages measurable improvements.<br>✓ Personalized goal setting to enhance engagement.<br>✓ Data-driven feedback from EMG and ultrasound for visible progress tracking.)	Methods Section → Recruitment (2.4)	
**7a.**	Detailed description of the decision rule(s) for determining exercise progression<br>✓ Participants progress based on biomechanical feedback, muscle activation improvements, and functional assessments.<br>✓ Increased intensity as training adaptations occur, ensuring neuromuscular optimization.	Methods Section → Main Core Training Protocol (3.3.2)	
**7b.**	Detailed description of how the exercise program was progressed<br>✓ Weeks 1-4 → Stability-focused drills with controlled movements.	Methods Section → Main Core Training Protocol (3.3.2)	

		✓ Weeks 5-8 → Power-based explosive exercises for advanced neuromuscular engagement.<br>✓ Modifications based on individual capacity and neuromuscular activation metrics.		
	**8.**	Detailed description of each exercise to enable replication (e.g. photographs, illustrations, video etc)<br>✓ Exercises documented via Kinovea software (angular velocity tracking).<br>✓ Biomechanical descriptions provided in supplementary materials.<br>✓ Images or visual aids included where needed.	Supplementary Materials	
	**9.**	Detailed description of any home program component (e.g. other exercises, stretching etc)<br>✓ No home program included → All exercises occur within supervised training facilities.	N/A	
	**10.**	Describe whether there are any non-exercise components (e.g. education, cognitive behavioural therapy, massage etc)<br>✓ Psychological assessment via SWLS questionnaire included to evaluate participant well-being.<br>✓ No additional interventions (e.g., massage, behavioral therapy).	Study Measuring Procedure (3.1)	
	**11.**	Describe the type and number of adverse events that occurred during exercise<br>✓ Monitored throughout study → Any injury or discomfort reported immediately.<br>✓ Standardized emergency response procedures in place.<br>✓ Expected risk profile remains minimal, given structured progression and supervision.	Methods Section → Study Setting (2.2)	
**WHERE: location**	**12.**	Describe the setting in which the exercises are performed<br>✓ Conducted at Aristotle University of Thessaloniki – Department of Physical Education & Sport Science (Serres).<br>✓ Fully equipped biomechanics lab for testing & assessments.<br>✓ Gyms designated for structured core training interventions.	Methods Section → Study Setting (2.2)	
**WHEN, HOW MUCH: dosage**	**13.**	Detailed description of the exercise intervention including, but not limited to, number of exercise repetitions/sets/sessions, session duration, intervention/program duration etc	Methods Section → Main Core Training Protocol (3.3.2)	

		✓ 8-week program with 3 training sessions per week (~60 min each session).<br>✓ Total of 24 structured training sessions.<br>✓ Exercise durations & intervals described in the protocol.		
**TAILORING: what, how**	**14a.**	Describe whether the exercises are generic (one size fits all) or tailored whether tailored to the individual<br>✓ Personalized adjustments → Adapted based on fitness level, neuromuscular responsiveness, and individual performance.	Methods Section → Study Measuring Procedure (3.1)	
	**14b.**	Detailed description of how exercises are tailored to the individual<br>✓ Beginners focus on core stability & controlled movement.<br>✓ Advanced participants progress to high-intensity explosive training.<br>✓ Tailored based on initial neuromuscular assessments.	Methods Section → Study Measuring Procedure (3.1)	
	**15.**	Describe the decision rule for determining the starting level at which people commence an exercise program (such as beginner, intermediate, advanced etc)<br>✓ Baseline assessments determine participant classification (Beginner vs. Advanced).<br>✓ Neuromuscular activation metrics guide initial progression.<br>✓ Participant comfort levels assessed before escalating intensity.	Methods Section → Study Measuring Procedure (3.1)	
**HOW WELL: planned, actual**	**16a.**	Describe how adherence or fidelity to the exercise intervention is assessed/measured<br>✓ Session attendance logs & compliance tracking.<br>✓ EMG & ultrasound assessments confirm neuromuscular activation progress.	Methods Section → Study Setting (2.2)	
	**16b.**	Describe the extent to which the intervention was delivered as planned<br>✓ Adherence will be formally evaluated post-study.<br>✓ Expected deviations (e.g., participant withdrawal) accounted for in sample size planning.	Study Measuring Procedure (3.1)	

*It is recommended that this checklist is used in conjunction with the Explanation and Elaboration Statement which is a guide each item in the CERT Checklist

The CERT Checklist is designed for reporting details of an exercise intervention. The CERT Checklist should be used in conjunction with a reporting checklist appropriate for the study type e.g. the CONSORT Statement (www.consort-statement.org) for randomised controlled trials, the SPIRIT Statement (www.spirit-statement.org) for a clinical trial protocol. For further guidance regarding reporting guidelines please consult the EQUATOR network

(www.equator-network.org)

** Authors – please use N/A if an item is not applicable Reviewers – please use ”?” if information is not provided or not/insufficiently reported

† If the information is not provided in the primary paper that is under consideration, please provide details of where this information is available e.g. in a published protocol, published papers (provide citation details) or on a website (provide the URL).

TIDieR
Template for Intervention Description and Replication

The TIDieR (Template for Intervention Description and Replication) Checklist*:

Information to include when describing an intervention and the location of the information

Item number	Item	Where located ** Primary paper (page or appendix number)	Other † (details)
	**BRIEF NAME**		
**1.**	Provide the name or a phrase that describes the intervention.	Structured Core Training Protocol for Muscle Activation, Hypertrophy, Balance, and Quality of Life in Physically Active Adults. ______________	______________
	**WHY**		
**2.**	Describe any rationale, theory, or goal of the elements essential to the intervention.	Core stability is fundamental to human movement, influencing postural control, injury prevention, and neuromuscular coordination. This intervention aims to:<br>✓ Enhance spinal stability, balance, and muscle strength<br>✓ Reduce musculoskeletal dysfunction risk, especially lower back pain<br>✓ Improve neuromuscular coordination for optimized functional movement<br>✓ Support public health initiatives in injury prevention & rehabilitation<br>____________	______________ _
	**WHAT**		

**3.**	Materials: Describe any physical or informational materials used in the intervention, including those provided to participants or used in intervention delivery or in training of intervention providers. Provide information on where the materials can be accessed (e.g. online appendix, URL).	Participants will engage in:<br>✓ Medicine balls for ballistic exercises<br>✓ Resistance bands for core stabilization drills<br>✓ EMG devices to measure muscle activation<br>✓ Ultrasound imaging for structural adaptations<br>✓ Balance platforms to assess postural control<br>✓ Biomechanical tracking software (Kinovea) for angular velocity assessments<br><br>Materials available in supplementary documents (e.g., methodology section, data collection protocol).<br><br>__________	____________<br>_
**4.**	Procedures: Describe each of the procedures, activities, and/or processes used in the intervention, including any enabling or support activities.	✓ Warm-up phase: Guided preparation for core activation<br>✓ Training phase: Eight-week progression from stability-focused drills to explosive movements<br>✓ Assessment phase: EMG, ultrasound, force plate testing, balance evaluation, kinematic analysis<br>✓ Psychological assessment: SWLS questionnaire<br><br>Procedures detailed in Methods section.<br><br>___________	____________<br>_
	**WHO PROVIDED**		
**5.**	For each category of intervention provider (e.g. psychologist, nursing assistant), describe their	✓ **Lead Researcher:** Ioannis Tsartsapakis (Ph.D. Exercise Science Specialist)	____________<br>_

	expertise, background and any specific training given.	✓ **Biomechanics & EMG Technicians:** Experts in neuromuscular assessments<br>✓ **Strength & Conditioning Coaches:** Specialized in core training interventions<br>____________	
	**HOW**		
**6.**	Describe the modes of delivery (e.g. face-to-face or by some other mechanism, such as internet or telephone) of the intervention and whether it was provided individually or in a group.	✓ Face-to-face supervised training in small-group sessions<br>✓ Individual assessments with real-time feedback (EMG & ultrasound imaging)<br>_____________	_____________<br>_
	**WHERE**		
**7.**	Describe the type(s) of location(s) where the intervention occurred, including any necessary infrastructure or relevant features.	✓ Conducted at Aristotle University of Thessaloniki – Department of Physical Education & Sport Science (Serres)<br>✓ Fully equipped biomechanics lab & exercise science facility<br>______________	_____________<br>_
	**WHEN and HOW MUCH**		
**8.**	Describe the number of times the intervention was delivered and over what period of time including the number of sessions, their schedule, and their duration, intensity or dose.	✓ 8-week intervention<br>✓ 3 sessions per week<br>✓ Each session: ~60 minutes (progressively increasing intensity)<br>______________	_____________<br>_
	**TAILORING**		

**9.**	If the intervention was planned to be personalised, titrated or adapted, then describe what, why, when, and how.	✓ Progressive overload system – modified for fitness level<br>✓ Stability training for beginners vs. explosive training for advanced<br>______________	______________ _
	**MODIFICATIONS**		
**10.‡**	If the intervention was modified during the course of the study, describe the changes (what, why, when, and how).	✓ N/A (Since this is a protocol submission, modifications will be documented post-study)<br>______________	______________ _
	**HOW WELL**		
**11.**	Planned: If intervention adherence or fidelity was assessed, describe how and by whom, and if any strategies were used to maintain or improve fidelity, describe them.	✓ Session logs to document participant attendance<br>✓ EMG & biomechanical tracking for adherence evaluation<br>✓ Expert supervision to ensure proper execution<br>______________	______________ _
**12.‡**	Actual: If intervention adherence or fidelity was assessed, describe the extent to which the intervention was delivered as planned.	N/A (Will be assessed post-study)<br>______________	______________ _

** **Authors** - use N/A if an item is not applicable for the intervention being described. **Reviewers** – use '?' if information about the element is not reported/not sufficiently reported.

† If the information is not provided in the primary paper, give details of where this information is available. This may include locations such as a published protocol or other published papers (provide citation details) or a website (provide the URL).

‡ If completing the TIDieR checklist for a protocol, these items are not relevant to the protocol and cannot be described until the study is complete.

* We strongly recommend using this checklist in conjunction with the TIDieR guide (see *BMJ* 2014;348:g1687) which contains an explanation and elaboration for each item.

* The focus of TIDieR is on reporting details of the intervention elements (and where relevant, comparison elements) of a study. Other elements and methodological features of studies are covered by other reporting statements and checklists and have not been duplicated as part of the TIDieR checklist. When a **randomised trial** is being reported, the TIDieR checklist should be used in conjunction with the CONSORT statement (see www.consort-statement.org) as an extension of **Item 5 of the CONSORT 2010 Statement.** When a **clinical trial protocol** is being reported, the TIDieR checklist should be used in conjunction with the SPIRIT statement as an extension of **Item 11 of the SPIRIT 2013 Statement** (see www.spirit-statement.org). For alternate study designs, TIDieR can be used in conjunction with the appropriate checklist for that study design (see www.equator-network.org).
