# Supplementary material for: Development and Implementation of a Core Training Protocol: Effects on Muscle Activation, Hypertrophy, Balance, and Quality of Life in Recreationally Active Adults
Source: Methods Protoc. 2025 Jul 8;8(4):77. doi: 10.3390/mps8040077 (PMC12286126; doi:10.3390/mps8040077)
Supplement: Supplementary file 1 [file mps-08-00077-s001.zip › Supplemetary files/mps-3708003 Concent form supplementary 3.pdf]

ΑΡΙΣΤΟΤΕΛΕΙΟ ΠΑΝΕΠΙΣΤΗΜΙΟ ΘΕΣΣΑΛΟΝΙΚΗΣ

ΤΕΦΑΑ ΣΕΡΡΩΝ

ΕΝΤΥΠΟ ΣΥΓΚΑΤΑΘΕΣΗΣ ΣΥΜΜΕΤΟΧΗΣ ΣΕ ΕΡΕΥΝΑ

Τίτλος έρευνας

«Ανάπτυξη και εφαρμογή ενός πρωτοκόλλου προπόνησης του κορμού: Επίδραση στην ενεργοποίηση των μυών, την υπερτροφία, την ισορροπία και την ποιότητα ζωής σε ενήλικες που δραστηριοποιούνται στην αναψυχή.»

Διαδικασία

Θα σας ζητηθεί να διαβάσετε οδηγίες συμπλήρωσης των ερωτηματολογίων της έρευνας. Η κατανόηση της διαδικασίας συμπλήρωση των ερωτηματολογίων και η συγκατάθεσή σας είναι απαραίτητες για να συμμετέχετε στην εργασία.

Αναμενόμενα οφέλη για εσάς και το κοινωνικό σύνολο

Για την πειραματική ομάδα:
Μέσω της συμμετοχής σας στην οκταεβδομαδιαία παρέμβαση προπόνησης του κορμού αναμένεται να έχετε σημαντικά οφέλη, βελτιώνοντας τη σταθερότητα της σπονδυλικής στήλης, την ισορροπία και τη μυϊκή ενδυνάμωση. Το πρόγραμμα θα ενισχύσει τη λειτουργική σταθερότητα, μειώνοντας τον κίνδυνο μυοσκελετικών δυσλειτουργιών και πόνου στη μέση, ενώ θα συμβάλει στην ανάπτυξη μυϊκής υπερτροφίας και κινητικής απόδοσης μέσω μετρήσιμων βελτιώσεων σε εκρηκτικές ασκήσεις. Η ενίσχυση της νευρομυϊκής συναρμογής και της κινητικής αίσθησης θα βελτιώσει την σωματική απόδοση και τις καθημερινές δραστηριότητες, ενώ η συνολική εμπειρία της προπόνησης θα συνεισφέρει θετικά στην ψυχολογική ευεξία, ενισχύοντας την αυτοπεποίθηση και τη συνολική ικανοποίηση από τη ζωή.

Για την ομάδα ελέγχου:
Η συμμετοχή σας είναι κρίσιμη για την επιστημονική ακρίβεια της μελέτης, καθώς θα μας βοηθήσετε να αξιολογήσουμε εάν το πρωτόκολλο προπόνησης κορμού δημιουργεί μετρήσιμες βελτιώσεις σε σύγκριση με άτομα που ακολουθούν πέρα από τη συνηθισμένη τους φυσική δραστηριότητα και ένα πρόγραμμα ενδυνάμωσης κοιλιακών και ραχιαίων. Κατά τη διάρκεια της έρευνας, θα υποβληθείτε σε αξιολογήσεις κινητικής απόδοσης, μυϊκής ενεργοποίησης και ψυχολογικής ευεξίας (EMG, υπερηχογραφία, δοκιμές ισορροπίας), συμβάλλοντας στη συλλογή σημαντικών δεδομένων για τη βελτιστοποίηση μελλοντικών προπονητικών παρεμβάσεων. Με τη συμμετοχή σας, συμβάλλετε άμεσα στην εξέλιξη της επιστήμης της άσκησης και της εξαγωγής έγκυρων συμπερασμάτων.

Οικονομική υποχρέωση / Πληρωμή για συμμετοχή

•Δεν χρειάζεται καμία οικονομική συμμετοχή από εσάς ή οποιονδήποτε ασφαλιστικό φορέα για τη συμμετοχή σας στην έρευνα

•Δεν θα λάβετε καμία αμοιβή για τη συμμετοχή σας στην έρευνα.

Ιδιωτικότητα και Εμπιστευτικότητα

Οι μόνοι άνθρωποι που θα γνωρίζουν ότι συμμετέχετε στην έρευνα είναι τα μέλη της ερευνητικής ομάδας. Καμία πληροφορία για εσάς ή πληροφορία που παρείχατε με τη συμμετοχή σας στην έρευνα δεν θα γίνει γνωστή σε άλλους χωρίς την έγγραφή συγκατάθεσή σας, με εξαίρεση:

•Όταν είναι απαραίτητο να προστατευτούν τα δικαιώματά σας ή για λόγους υγείας (για παράδειγμα, σε περίπτωση τραυματισμού και ανάγκης για διακομιδή σε νοσοκομείο), ή

•Εάν απαιτείται από το νόμο.

Όταν τα αποτελέσματα της εργασίας δημοσιευτούν ή ανακοινωθούν σε συνέδρια, καμία πληροφόρηση δεν θα φανερώνει την ταυτότητά σας.

Συμμετοχή και Αποχώρηση

Η συμμετοχή σας στην έρευνα είναι ΕΘΕΛΟΝΤΙΚΗ. Εάν επιλέξετε να μην συμμετέχετε, τότε δεν επηρεάζεται η σχέση σας με το πανεπιστήμιο, ή με τους ερευνητές ούτε επηρεάζεται το δικαίωμά σας για όποιες άλλες υπηρεσίες τις οποίες δικαιούστε. Εάν αποφασίσετε να συμμετέχετε, είστε ελεύθερος /η να αποχωρήσετε όποτε θέλετε χωρίς καμία δέσμευση ή επίπτωση. Για οποιαδήποτε ερώτηση σχετικά με τα δικαιώματά σας από τη συμμετοχή σας ως υποκείμενο σε ερευνητική εργασία, μπορείτε να απευθυνθείτε στην Ελληνική Οργάνωση για τα δικαιώματα των υποκειμένων σε έρευνα.

Απόσυρση της συμμετοχής με απόφαση του ερευνητή

Οι ερευνητές έχουν δικαίωμα να σας ζητήσουν να μη συμμετέχετε στην έρευνα, εάν το επιβάλλουν οι συνθήκες.

Υπεύθυνοι επικοινωνίας

Για οποιαδήποτε πληροφορία επικοινωνήστε με: Τσαρτσαπάκης Ιωάννης : 6982906442

Υπογραφή του συμμετέχοντα στην έρευνα ή νόμιμου εκπροσώπου του

Διάβασα ή κάποιας άλλος μου ανάγνωσε, και κατανοώ πλήρως τις πληροφορίες, οι οποίες αναγράφονται σε αυτήν τη φόρμα. Μου δόθηκε η ευκαιρία να υποβάλλω ερωτήσεις και όλες οι ερωτήσεις απαντήθηκαν πλήρως. Έλαβα αντίγραφο αυτής της φόρμας όπως και των δικαιωμάτων των συμμετεχόντων ατόμων στην έρευνα.

Μόνο για γυναίκες συμμετέχουσες σε συγκεκριμένα διαγνωστικά πρωτόκολλα:

Βεβαιώνω ότι γνωρίζω πως δεν είμαι έγκυος και ότι ερωτήθηκα εάν υπάρχει τέτοια πιθανότητα.

Υπογράφοντας τον παρόν έντυπο, συμφωνώ να συμμετέχω στη συγκεκριμένη έρευνα.

Ονοματεπώνυμο_________________________________ Ημερομηνία:____________

Υπογραφή του ερευνητή

Έχω εξηγήσει την έρευνα στον /στην συμμετέχοντα /ουσα ή στο νόμιμο εκπρόσωπό του /της και απάντησα σε όλες τις ερωτήσεις του/της. Θεωρώ ότι καταλαβαίνει τις πληροφορίες που αναγράφονται στο συγκεκριμένο έντυπο και συμμετέχει στη συγκεκριμένη έρευνα με την ελεύθερη του/της βούληση.

Ονοματεπώνυμο __________________________________ Ημερομηνία ___________

ΠΑΡΑΡΤΗΜΑ: Πρωτόκολλο ερευνητικής διαδικασίας

Αρχικά θα σας εξηγήσουμε την όλη διαδικασία του πρωτοκόλλου μέτρησης.

Σωματομετρικά στοιχεία: Συμπληρώστε μόνο την ηλικία και τους τρόπους που ασκήστε (τρέξιμο, ποδηλασία, άσκηση στο γυμναστήριο, πεζοπορία βουνού, κολύμβηση), πόσα χρόνια ασκείστε, πόσες φορές την εβδομάδα και πόση ώρα. Το βάρος και ύψος θα μετρηθούν με αξιόπιστα όργανα από τους ειδικούς επιστήμονες που είναι υπεύθυνοι για την έρευνα.

Ζέσταμα: με την καθοδήγηση των ερευνητών θα κάνετε ένα ζέσταμα για να προετοιμάσετε τους μύες σας για την άσκηση «Άνωθεν ρίψη της ιατρικής μπάλας». Κατόπιν θα κάνετε μερικές δοκιμαστικές ρίψεις για να τελειοποιήσετε την τεχνική σας.

Πρωτόκολλο Εκτέλεσης & Μετρήσεων

Οι συμμετέχοντες θα τοποθετηθούν σε σταθερή επιφάνεια με τα πόδια στο εύρος των ώμων και το βάρος κατανεμημένο ομοιόμορφα. Η άσκηση θα εκτελεστεί σε δύο επαναλήψεις, με δύο δοκιμαστικές ρίψεις προηγουμένως για την εξοικείωση με την τεχνική.

1. Στάση & Προετοιμασία

- Κράτημα της ιατρικής μπάλας στο επίπεδο του θώρακα, με συμμετρική λαβή.

- Ενεργοποίηση του κορμού με σταθεροποίηση της σπονδυλικής στήλης για μέγιστη απόδοση.

- Βαθιά εισπνοή για αύξηση της ενδοκοιλιακής πίεσης πριν τη ρίψη.

2. Εκτέλεση Άσκησης

- Ανύψωση της μπάλας πάνω από το κεφάλι με πλήρη έκταση των άνω άκρων.

- Εκρηκτική ρίψη της μπάλας στο δυναμόμετρο με ταυτόχρονη εκπνοή και ενεργοποίηση του κορμού.

- Έλεγχος κίνησης ώστε η μπάλας να κατευθύνεται ευθεία προς την πλατφόρμα μέτρησης.

3. Μετρήσεις κατά την εκτέλεση

🔹 Δύναμη πρόσκρουσης μέσω του Kistler 3D δυναμόμετρου.

🔹 Ηλεκτρομυογραφική (EMG) καταγραφή για την ενεργοποίηση των μυών ορθού κοιλιακού, εξωτερικού λοξού και ιερονωτιαίου μυ.

🔹 Βιομηχανική ανάλυση μέσω Kinovea software για τη γωνιακή ταχύτητα των ώμων, ισχίων και γονάτων.

🔹 Υπερηχογραφική μέτρηση του πάχους των κοιλιακών μυών πριν και μετά την άσκηση για αξιολόγηση της μυϊκής ενεργοποίησης.

🔹 Ψυχολογική αξιολόγηση μέσω SWLS ερωτηματολογίου μετά την ολοκλήρωση των δοκιμών.

Μετά την ολοκλήρωση των μετρήσεων, οι συμμετέχοντες θα έχετε μια σύντομη περίοδο επαναφοράς και θα λάβετε οδηγίες σχετικά με την καταγραφή των αποτελεσμάτων σας. Α ν δεν καταλαβαίνετε κάποια φράση ή διαδικασία ρωτήστε τον ερευνητή για διευκρινήσεις.

ΙΩΑΝΝΗΣ, I’ve got you covered! 🚀 Here’s the **English translation** of your informed consent document, ensuring clarity, professionalism, and alignment with ethical standards:

Aristotle University of Thessaloniki

Department of Physical Education & Sport Science – Serres
Informed Consent Form for Research Participation

Research Title:

" Development and Implementation of a Core Training Protocol: Effects on Muscle Activation, Hypertrophy, Balance, and Quality of Life in Recreationally Active Adults."

Study Procedure:

Participants will be asked to read instructions regarding the completion of study questionnaires. Understanding the questionnaire procedure and providing informed consent is required for participation in the study.

Expected Benefits for You and the Community:

For the Experimental Group:

Participation in the **eight-week core training intervention** is expected to provide **significant benefits**, including:
✓ Enhanced spinal stability, balance, and muscle strength.
✓ Reduction in the risk of musculoskeletal dysfunctions and lower back pain.
✓ Measurable improvements in muscle hypertrophy and motor performance in explosive exercises.
✓ Better neuromuscular coordination and kinesthetic awareness, improving physical function and daily activities.
✓ Positive psychological effects, including increased self-confidence and overall life satisfaction.

For the Control Group:

Your participation is crucial for the **scientific accuracy** of the study, as it will allow us to determine whether the **core training protocol leads to measurable improvements** compared to individuals maintaining their usual physical activity plus an abs and back strength program.
✓ You will undergo motor performance assessments, muscle activation testing, and psychological well-being evaluations (EMG, ultrasound scans, balance tests).
✓ Your involvement will contribute to data collection for optimizing future training interventions.
✓ By participating, you actively support the advancement of exercise science and help generate valuable scientific conclusions.

Financial Obligation / Compensation:

- **No financial participation is required**—neither from you nor any insurance provider.
- ❌ **You will not receive financial compensation** for participation.

Privacy and Confidentiality:

✓ **Only members of the research team will be aware of your participation.**
✓ No personal information or data provided in the study will be shared without your written consent, except in cases where:

- It is necessary to protect your **rights or health** (e.g., injury requiring medical attention).
- Disclosure is **legally required**.
  ✓ **Results will be published anonymously**—your identity will never be revealed in scientific presentations or journal publications.

Participation and Withdrawal:

✓ **Your participation is voluntary.** If you choose **not** to participate, your relationship with the university, researchers, or access to any entitled services **will not be affected**.
✓ You are **free to withdraw at any time** without obligation or consequence.
✓ If you have concerns about your **rights as a research participant**, you may contact the **Greek Organization for Research Participant Rights**.

Withdrawal by Researcher Decision:

The research team may request withdrawal if study conditions require it.

Contact Information:

For any inquiries, contact **Ioannis Tsartsapakis**: +30 6982906442

Participant's Signature:

📄 **Statement of Understanding**
"I have read (or had someone read to me) and fully understand the information provided in this form. I have had the opportunity to ask questions, and all questions have been answered completely. I have received a copy of this form, as well as information about my rights as a research participant."

For female participants in specific diagnostic protocols:
"I confirm that I am not pregnant and have been asked whether pregnancy is a possibility."

By signing this document, I voluntarily agree to participate in the study.

Full Name: ___________________________

Date: ___________

Participant Signature: ___________________________

Researcher Signature:
"I have explained the study to the participant or their legal representative, answered all their questions, and confirmed that they understand the information provided. I believe they are participating voluntarily."

Full Name: ___________________________

Date: ___________

Appendix: Research Protocol Procedure

1. Initial Instructions & Physical Data Collection:

Participants will complete basic demographic information (age, type of physical activity, years of training, frequency per week, duration per session).
Weight and height will be measured by qualified scientists using validated instruments.

2. Warm-Up & Familiarization:

Guided warm-up exercises will be performed to prepare muscles for the "Overhead Medicine Ball Throw."
Participants will perform test throws to refine technique before measurements begin.

3. Execution & Measurement Protocol:

Stable stance: Feet shoulder-width apart, weight evenly distributed.
Holding the medicine ball: Chest level, symmetrical grip.
Core engagement: Spinal stabilization for maximum performance.
Deep inhale: Increases intra-abdominal pressure before the throw.
Explosive throw: Overhead motion with full arm extension.
Controlled release: Ensuring accurate trajectory toward measurement platform.

4. Scientific Assessments:

Force impact measurement via Kistler 3D dynamometer.
Electromyography (EMG) readings for rectus abdominis, external oblique, and erector spinae muscles.
Biomechanical analysis using Kinovea software for angular velocity in shoulders, hips, and knees.
Ultrasound imaging to measure pre- and post-exercise core muscle thickness.
Psychological assessment via SWLS questionnaire post-intervention.

5. Post-Assessment & Data Collection:

Recovery period following measurement protocol.
Final participant instructions for recording results.
If clarification is needed, participants may ask the researcher questions.
