# Supplementary material for: Development and Implementation of a Core Training Protocol: Effects on Muscle Activation, Hypertrophy, Balance, and Quality of Life in Recreationally Active Adults
Source: Methods Protoc. 2025 Jul 8;8(4):77. doi: 10.3390/mps8040077 (PMC12286126; doi:10.3390/mps8040077)
Supplement: Supplementary file 1 [file mps-08-00077-s001.zip › Supplemetary files/mps-3708003 Protocol exercises supplementary 1.pdf]

Supplementary file 1.

Supplementary File: 8th week intervention Core Training, Exercise Instructions

The subsequent section delineates the meticulous execution guidelines for the warm-up, main core training protocol and cool down exercises, ensuring that each movement is articulated with precision and scientific rigor, in accordance with the CERT & TIDieR frameworks. All exercises are presented in detail in the supplementary video file.

A) Warm-up Protocol for Core Training (Approx. 6 min)

1) **Suitcase Walk (Forward/Backward)**

   Setup: Hold a dumbbell or kettlebell in both hands, keeping your body upright with a neutral spine.
   Execution: Walk 15 meters forward, maintaining core tension to resist lateral bending. Then walk 15 meters backward, engaging posterior chain activation.
   Breathing: Exhale during steps and brace the core throughout.
   Common Mistakes: Avoid leaning toward the weighted side or overarching the lower back.

2) **Overhead Dumbbells Walk**

   Setup: Hold light dumbbells overhead with elbows locked and shoulders engaged.
   Execution: Walk 15 meters forward and 15 meters backward, maintaining overhead stability and engaging the core and scapular stabilizers.
   Breathing: Controlled diaphragmatic breathing, with exhales synchronized with each step.
   Common Mistakes: Avoid excessive lumbar extension or shrugging shoulders upward.

3) **Walking 5m + High Knee Jog**

   Setup: Stand tall with feet hip-width apart.
   Execution: Walk 5 meters forward, then transition into a 10-meter high-knee jog, ensuring core engagement and dynamic stability.
   Breathing: Inhale during transitions, exhale while driving knees upward.
   Common Mistakes: Avoid leaning backward or lifting knees passively.

4) **Bend Over T-Rotation**

   Setup: Assume a hip-hinge position, hands extended forward.
   Execution: Rotate one arm upward, following with torso rotation, then alternate sides for 30 seconds. Maintain neutral spinal alignment.
   Breathing: Exhale during rotations, maintaining ribcage expansion.
   Common Mistakes: Avoid excessive twisting in lumbar spine or shrugging shoulders.

5) **Squat Lateral Reach + Squat Side Reach**

   Setup: Stand in a wide squat stance, arms extended.
   Execution: Perform a deep squat, reaching laterally with one arm, then transition into a side reach squat, alternating sides for 45 seconds.
   Breathing: Exhale during downward phases, inhale while resetting.

Common Mistakes: Avoid excessive knee valgus (inward collapsing).

6) **Lying Bend Leg Twist**

Setup: Lie supine, knees bent at 90 degrees, arms extended.
Execution: Rotate knees toward one side, return to center, then alternate for 30 seconds, controlling spinal rotation.
Breathing: Exhale during twists, inhale during resets.
Common Mistakes: Avoid lifting shoulders off the ground.

7) **Bridge Exercise**

Setup: Lie supine, feet planted, knees bent.
Execution: Drive hips upward and downward for 30 seconds, try to keep neutral pelvis position.
Breathing: Exhale while lifting, inhale while lowering.
Common Mistakes: Avoid overextending lumbar spine.

8) **Knee Push-ups**

Setup: Position yourself in modified push-up stance, knees grounded.
Execution: Lower body with elbows at 45 degrees, maintaining core tension, repeat for 30 seconds.
Breathing: Exhale while pushing up, inhale while lowering.
Common Mistakes: Avoid arching lower back or flaring elbows excessively.

B) Main Core Training Protocol (Approx. 30 - 32 min)

Triad 1 – Stability, Strength & High-Intensity Core Power Exercises

1) Squat + Cross Knees to Elbows *(Stability Exercise – 45 sec)*

Setup: Stand with feet hip-width apart, hands behind your head.
Execution: Perform a deep squat, then as you stand, lift one knee toward the opposite elbow, engaging the obliques. Alternate sides.
Breathing: Exhale during knee drive, inhale while squatting.
Common Mistakes: Avoid rounding the back or rushing the movement.

2) Bird Dog Exercise *(Strength Exercise – 45 sec)*

Setup: Get into a quadruped position (hands and knees).
Execution: Extend one arm and opposite leg simultaneously while maintaining core stability, then switch sides.
Breathing: Inhale during extension, exhale while bringing limbs back in.
Common Mistakes: Avoid arching the lower back or lifting limbs too high.

3) Three Repeat Ice Skater + One High Jump *(High-Intensity Core Power Exercise– 45 sec)*

Setup: Start in an athletic stance.

Execution: Perform three lateral ice skater jumps, then explode upward with a high vertical jump, landing softly with bend knees. Repeat sequence.
Breathing: Exhale during explosive movements, inhale during resets.
Common Mistakes: Avoid landing stiffly or losing balance during lateral movement.

Triad 2 – Stability, Strength & High-Intensity Core Power Exercises

1) Standing to Walkout Plank *(Stability Exercise – 45 sec)*

Setup: Stand with feet hip-width apart, arms relaxed at sides.
Execution: Hinge at the hips and walk hands forward into a full plank position, hold briefly, then reverse the motion back to standing.
Breathing: Inhale while lowering, exhale while pushing back up.
Common Mistakes: Avoid sagging hips or rushing the movement—maintain controlled pacing.

2) Dead Bug Exercise *(Strength Exercise – 45 sec)*

Setup: Lie on your back, arms extended above shoulders, knees bent at 90 degrees.
Execution: Lower one arm and opposite leg toward the ground while keeping spine neutral, then return to start and alternate.
Breathing: Exhale while lowering limbs, inhale while returning.
Common Mistakes: Avoid arching lower back—maintain deep core engagement.

3) Side to Side Lateral Floor Tap (3m) *(High-Intensity Core Power Exercise – 45 sec)*

Setup: Stand in athletic stance, feet shoulder-width apart.
Execution: Quickly shuffle laterally for 3 meters, tapping the floor at each side before changing direction.
Breathing: Exhale during side-to-side movement, inhale during transitions.
Common Mistakes: Avoid locking knees—keep dynamic movement mechanics activated.

Triad 3 – Stability, Strength & High-Intensity Core Power Exercises

1) Standing Dumbbell Windmill *(Stability Exercise – 45 sec)*

Setup: Stand with feet wider than shoulder-width apart, holding a light dumbbell in one hand, extended overhead.
Execution: Slowly hinge at the hip while lowering the opposite hand toward the foot, maintaining core engagement and shoulder stability. Return to start and switch sides.
Breathing: Inhale while lowering, exhale during return.
Common Mistakes: Avoid rounding the back or losing dumbbell control—maintain strict form.

2) Sit-ups *(Strength Exercise – 45 sec)*

Setup: Lie supine, knees bent, feet planted.

Execution: Engage abdominals and sit up smoothly, bringing hands toward the knees, then slowly lower back down.
Breathing: Exhale while sitting up, inhale during descent.
Common Mistakes: Avoid pulling on the neck—focus on core engagement.

3) Lateral Step Jump with Up-Down Exercise *(High-Intensity Core Power Exercise – 45 sec)*

Setup: Stand in athletic stance, feet hip-width apart.
Execution: Perform a lateral jump, landing softly, then drop into a quick up-down (burpee-style movement). Repeat.
Breathing: Exhale during jumps, inhale during resets.
Common Mistakes: Avoid landing stiffly—maintain smooth transitions.

Triad 4 – Stability, Strength & High-Intensity Core Power Exercises

1) Side to Side Beast Crawl *(Stability Exercise – 45 sec)*

Setup: Start in a quadruped position, with knees hovering slightly above the ground and hands aligned under shoulders.
Execution: Move laterally by stepping one hand and opposite foot to the side, maintaining low core tension and neutral spine alignment. Continue side-to-side movement.
Breathing: Exhale during lateral steps, inhale during resets.
Common Mistakes: Avoid lifting hips too high or losing core engagement—keep movement controlled.

2) Reverse Crunch Abs *(Strength Exercise – 45 sec)*

Setup: Lie supine, knees bent at 90 degrees, hands by sides.
Execution: Engage lower abdominals and lift hips off the ground, bringing knees toward chest, then lower back slowly.
Breathing: Exhale while lifting hips, inhale during descent.
Common Mistakes: Avoid using momentum—focus on strict core control.

3) Jack Squat + Twist Jump *(High-Intensity Core Power Exercise – 45 sec)*

Setup: Stand with feet hip-width apart.
Execution: Perform a squat, then explode upward, twisting 90 degrees mid-air before landing. Alternate twist directions.
Breathing: Exhale during jumps, inhale during reset.
Common Mistakes: Avoid uneven landings—control descent.

Triad 5 – Stability, Strength & High-Intensity Core Power Exercises

1) Front Lunge Body Twist *(Stability Exercise – 45 sec)*

Setup: Stand tall with feet hip-width apart, holding hands together at chest level.

Execution: Step forward into a deep lunge, then rotate the torso toward the forward leg while maintaining core engagement. Return and switch sides.
Breathing: Exhale during rotation, inhale while resetting.
Common Mistakes: Avoid leaning forward excessively—maintain upright posture.

2) Alternative Side Plank *(Strength Exercise – 45 sec)*

Setup: Begin in a side plank position, supporting body weight on one forearm and foot.
Execution: Hold for a few seconds, then switch to the opposite side, ensuring neutral spine alignment and core engagement.
Breathing: Slow controlled breathing, inhale while transitioning, exhale while maintaining position.
Common Mistakes: Avoid hip sagging—engage obliques fully.

3) Running 3m Forward & Backward with Floor Tap *(High-Intensity Core Power Exercise – 45 sec)*

Setup: Stand ready in an athletic stance.
Execution: Sprint 3 meters forward, touch the floor, then quickly reverse direction and run backward. Repeat continuously.
Breathing: Exhale during sprints, inhale during transitions.
Common Mistakes: Avoid stopping abruptly—focus on smooth directional changes.

Triad 6 – Stability, Strength & High-Intensity Core Power Exercises

1) Downward Dog to Cobra *(Stability Exercise – 45 sec)*

Setup: Begin in a Downward Dog position, with hips raised, hands shoulder-width apart, and heels grounded.
Execution: Slowly transition into Cobra pose, lowering hips toward the ground while extending the chest forward. Hold briefly, then return to Downward Dog.
Breathing: Inhale while lowering, exhale while pushing hips back up.
Common Mistakes: Avoid collapsing the lower back—engage core throughout.

2) Superman Exercise with Arm & Leg Lateral Extension *(Strength Exercise – 45 sec)*

Setup: Lie prone, arms extended forward.
Execution: Lift arms and legs simultaneously, then extend arms and legs laterally before returning to center. Lower back down and repeat.
Breathing: Exhale during lifts, inhale during resets.
Common Mistakes: Avoid hyperextending the neck—keep gaze neutral.

3) Lateral 3 Steps High Knees Shuffle *(High-Intensity Core Power Exercise – 45 sec)*

Setup: Stand in athletic stance, feet hip-width apart.
Execution: Perform three quick lateral steps, then drive knees high before repeating in the opposite direction.
Breathing: Exhale during lateral movement, inhale while resetting.

Common Mistakes: Avoid sluggish knee drive—maintain explosive movement mechanics.

Triad 7 – Stability, Strength & High-Intensity Core Power Exercises

1) Prisoner Get-Up Exercise *(Stability Exercise – 45 sec)*

Setup: Stand with feet hip-width apart, hands placed behind the head.
Execution: Lower into a kneeling position, maintaining upright posture, then rise back to standing without using hands for support.
Breathing: Exhale while standing up, inhale during descent.
Common Mistakes: Avoid rushing the movement—maintain core activation and controlled transitions.

2) Front Plank Moving Dumbbell Left-Right *(Strength Exercise – 45 sec)*

Setup: Get into a forearm plank position, a dumbbell placed slightly in front of you.
Execution: Using one hand, move the dumbbell laterally from one side to the other, engaging core stability and anti-rotation control.
Breathing: Exhale while moving the dumbbell, inhale while resetting.
Common Mistakes: Avoid hip swaying—keep spine neutral and core tight.

3) Burpee Broad Jump *(High-Intensity Core Power Exercise – 45 sec)*

Setup: Stand in an athletic stance, feet shoulder-width apart.
Execution: Perform a burpee, then explode forward into a broad jump, landing softly. Repeat the sequence continuously.
Breathing: Exhale during explosive jumps, inhale during resets.
Common Mistakes: Avoid stiff landings—focus on shock absorption and fluid movement.

Triad 8 – Stability, Strength & High-Intensity Core Power Exercises

1) Thruster with Dumbbell *(Stability Exercise – 45 sec)*

Setup: Hold dumbbells at chest level, feet hip-width apart.
Execution: Perform a deep squat, then explode upward, pressing dumbbells overhead.
Breathing: Exhale during overhead press, inhale during squat descent.
Common Mistakes: Avoid pressing too early—use leg drive efficiently.

2) Russian Twist with Dumbbell *(Strength Exercise – 45 sec)*

Setup: Sit on the floor, holding a dumbbell, knees bent and feet elevated.
Execution: Rotate torso side to side, touching the dumbbell to the floor on each side.
Breathing: Exhale during each twist, inhale while resetting.
Common Mistakes: Avoid using arm movement instead of true torso rotation—focus on core engagement.

3) Explosive Jumping Alternating Lunges *(High-Intensity Core Power Exercise – 45 sec)*

Setup: Stand with feet hip-width apart.
Execution: Jump explosively into a forward lunge, switching legs in mid-air, landing smoothly.
Breathing: Exhale during jumps, inhale while resetting.
Common Mistakes: Avoid unstable landings—keep core braced throughout.

C) Stretching & Cool-Down Protocol (Approx. 5 min)

1) Standing Rolling Down Stretch – 20 sec

Setup: Stand tall, feet hip-width apart.
Execution: Slowly roll down vertebra by vertebra, reaching for the ground, feeling the stretch through the hamstrings and spine.
Breathing: Exhale during descent, inhale while rolling back up.
Common Mistakes: Avoid rushing back up—ensure smooth spinal articulation.

2) Hip Flexor Stretches (Alternating Sides) – 20 sec each side

Setup: Step into a deep lunge with one leg forward, keeping the back knee lightly grounded.
Execution: Press hips forward, keeping spine neutral, to stretch the hip flexors and quadriceps. Hold the position, then switch sides.
Breathing: Inhale while setting up, exhale to deepen the stretch.
Common Mistakes: Avoid arching the lower back excessively—focus on keeping core engaged.

3) Cat-Camel Stretches – 20 sec

Setup: Start in a quadruped position (on hands and knees).
Execution: Alternate between arching (cat) and rounding (camel) the spine, moving smoothly through the sequence.
Breathing: Inhale during arching, exhale during rounding.
Common Mistakes: Avoid rushing the movement—focus on gradual mobility enhancement.

4) Threading the Needle Back Stretch – 40 sec

Setup: Begin in a quadruped position, arms extended.
Execution: Rotate one arm under the torso, reaching across, stretching the upper back **and shoulders**. Hold briefly, then switch sides.
Breathing: Exhale deeply into the stretch, inhale while returning to start.
Common Mistakes: Avoid collapsing into the stretch—maintain controlled movements.

5) Child's Pose Stretch – 20 sec

Setup: Kneel on the floor, toes pointing back, arms extended forward.
Execution: Sit back onto heels, press chest downward, extending arms for spinal decompression.
Breathing: Slow diaphragmatic breathing, with long exhalations.
Common Mistakes: Avoid overreaching the shoulders—keep movement relaxed.

6) Scorpion Stretch (Alternating Sides) – 20 sec each side

Setup: Lie prone, arms extended to the sides.
Execution: Lift one leg and cross it over the body, twisting the lower back gently. Return to start, then switch sides.
Breathing: Exhale while rotating, inhale while resetting.
Common Mistakes: Avoid over-rotating—focus on gentle mobility enhancement.

7) Cobra Extension Stretch – 20 sec

Setup: Begin prone, hands placed near the shoulders.
Execution: Extend arms, lifting chest upward, stretching the abdominals and spine.
Breathing: Deep inhalation during extension, slow exhalation for relaxation.
Common Mistakes: Avoid hyperextending the neck—keep gaze forward.

For a visual representation of the exercises, you can watch the video at the link below:

https://www.youtube.com/watch?v=ql8SD07abnI&t=65s

Control Group: Standardized two-day-per-week abs and back exercise routine.

Recommended 20-25 Min Abs & Back Routine for Control Group

Structure: 2 Sessions Per Week
Focus: Core endurance & spinal health without interference with the study's intervention

Warm-Up (5 min)

- Cat-Cow Stretch (30 – 40 sec) – Mobilizes the spine & enhances flexibility
- Standing Arm Circles (30 – 40 sec) – Loosens shoulders & upper back
- Dead Bug Activation (45 – 50 sec) – Prepares core muscles for engagement
- Glute Bridge Hold (45 – 50 sec) Activates posterior chain
- Standing Torso Twist (30 – 40 sec) - Activates the core & spinal mobility

Main Workout (15 min)

Perform two rounds of the following exercises:

- Front Plank Hold – 30 – 40 sec
- Side Plank (each side) – 20 – 40 sec

- Bird Dog Exercise – 8 – 10 reps per side
- Reverse Crunches – 12 - 15 reps
- Superman Hold – 30 – 40 sec
- Seated Back Extension Stretch – 20 – 30 sec

Cool Down (5 min)

- Child’s Pose Stretch (20 -30 sec)
- Cobra Stretch (20 – 30 sec)
- Lower Back Twist Stretch (20 – 30 sec per side)
- Seated Forward Fold Stretch (20 – 30 sec)
- Standing Side Stretch (20 – 30 sec per side)

Control Group Exercise Instructions

The subsequent section delineates meticulous execution protocols for the standardized two-day-per-week abs and back routine allocated to the control group. The exercises have been meticulously designed to ensure that patients receive a comprehensive core endurance and spinal health workout, while circumventing the potential adverse effects that could result from direct replication of the intervention protocol.

A) Warm-Up Protocol (Approx. 5 min)

1) Cat-Cow Stretch (30 -40 sec)
Setup: Begin in a quadruped position (on hands and knees).
Execution: Alternate between arching the spine (cat) and rounding it (cow) in a controlled manner.
Breathing: Inhale during spinal extension, exhale while rounding the back.
Common Mistakes: Avoid rushing—focus on smooth mobility and core engagement.

2) Standing Arm Circles (30 - 40 sec)
Setup: Stand tall, feet hip-width apart, arms extended at shoulder level.
Execution: Perform small controlled circles forward for 15 sec, then reverse for 15 sec.
Breathing: Maintain deep, steady breathing throughout.
Common Mistakes: Avoid shrugging shoulders—focus on fluid movement.

3) Dead Bug Activation (45 - 50 sec)
Setup: Lie supine, arms extended above shoulders, knees bent at 90 degrees.
Execution: Lower one arm and opposite leg toward the ground, then return to start; alternate sides.
Breathing: Exhale while lowering limbs, inhale while returning.
Common Mistakes: Avoid arching the lower back—keep deep core engaged.

4) Glute Bridge Hold (45 -50 sec)
Setup: Lie supine, knees bent, feet planted.
Execution: Lift hips toward the ceiling, holding a **neutral pelvis position**.
Breathing: Exhale while lifting, inhale while maintaining position.
Common Mistakes: Avoid excessive lumbar extension—focus on **glute activation**.

5) Standing Torso Twist (30 – 40 sec)
Setup: Stand tall, feet hip-width apart, arms bent at 90 degrees, hands positioned at chest level.
Execution: Rotate the torso side to side, engaging obliques and maintaining a controlled pace. Keep hips stable to isolate upper-body movement.
Breathing: Exhale during rotation, inhale while resetting to center.
Common Mistakes: Avoid overextending the twist—movement should be controlled and initiated by the core, not the arms.

B) Main Workout Routine (Approx. 15 min)

Perform two rounds of the following exercises:

1) Front Plank Hold (30 – 40 sec)
Setup: Get into a forearm plank position, feet hip-width apart.
Execution: Maintain full-body tension while keeping a neutral spine.
Breathing: Slow, controlled breathing.
Common Mistakes: Avoid hip sagging or shoulder tension—keep engaged.

2) **Side Plank (20-30 sec per side)**
Setup: Start in a side plank position, supporting weight on one forearm and foot.
Execution: Hold for duration, then switch sides.
Breathing: Maintain steady breath control throughout.
Common Mistakes: Avoid hip dipping—focus on oblique stability.

3) **Bird Dog Exercise (8 -10 reps per side)**
Setup: Begin in a quadruped position.
Execution: Extend one arm and opposite leg simultaneously, hold briefly, then switch.
Breathing: Inhale during extension, exhale when returning.
Common Mistakes: Avoid overextending the lower back—keep core braced.

4) Reverse Crunches (12 - 15 reps)
Setup: Lie supine, knees bent at 90 degrees.
Execution: Lift hips off the ground, bringing knees toward the chest, then lower slowly.
Breathing: Exhale during lifting, inhale during descent.
Common Mistakes: Avoid using momentum—focus on strict core control.

5) **Superman Hold (30 – 40 sec)**
Setup: Lie prone, arms extended forward.
Execution: Simultaneously lift arms, chest, and legs off the ground, hold position.

Breathing: Exhale during lift, inhale while holding.
Common Mistakes: Avoid hyperextending the neck—keep gaze neutral.

6) **Seated Back Extension Stretch (20 - 30 sec)**
Setup: Sit on the floor, legs extended forward.
Execution: Lean forward, reaching toward toes while keeping spine elongated.
Breathing: Deep inhales and slow exhales.
Common Mistakes: Avoid rounding the back excessively—focus on gradual stretch.

C) Cool Down Routine (Approx. 5 min)

1) Child's Pose Stretch (20 – 30 sec)
Setup: Kneel on the floor, arms extended forward.
Execution: Sit back onto heels, pressing chest downward.
Breathing: Long exhalations for relaxation.
Common Mistakes: Avoid shoulder tension—keep movement natural.

2) Cobra Stretch (20 – 30 sec)
Setup: Lie prone, hands placed near shoulders.
Execution: Extend arms, lifting chest upward, stretching abdominals and spine.
Breathing: Deep inhale on extension, slow exhale to relax.
Common Mistakes: Avoid hyperextending neck—keep gaze forward.

3) Lower Back Twist Stretch (20 – 30 sec per side)
Setup: Lie supine, knees bent.
Execution: Rotate knees toward **one side**, holding briefly, then switch.
Breathing: Exhale deeply during rotation.
Common Mistakes: Avoid lifting shoulders off the ground—keep movement controlled.

4) Seated Forward Fold Stretch (20 – 30 sec)
Setup: Sit with legs extended forward, toes pointing up.
Execution: Slowly lean forward, reaching toward the toes while keeping the spine elongated.
Breathing: Inhale to lengthen the spine, exhale to deepen the stretch.
Common Mistakes: Avoid rounding the back excessively—keep movement gradual and controlled.

5) Standing Side Stretch (20 – 30 sec per side)
Setup: Stand tall, feet hip-width apart, arms extended overhead.
Execution: Gently lean to one side, feeling the stretch along the obliques and lower back. Hold briefly, then switch.
Breathing: Exhale while stretching, inhale while returning to center.
Common Mistakes: Avoid collapsing forward—keep posture upright throughout.

participant flowchart Supplementary Figure S1.

Participants recruited

↓

Screening for eligibility

↓

Informed consent obtained

↓

Random allocation

↙ ↘

Experimental Control

Group Group

↓ ↓

Baseline Testing Baseline Testing

↓ ↓

Intervention No Intervention

↓ ↓

Post-Testing Post-Testing
