# Supplementary material for: Development and Implementation of a Core Training Protocol: Effects on Muscle Activation, Hypertrophy, Balance, and Quality of Life in Recreationally Active Adults
Source: Methods Protoc. 2025 Jul 8;8(4):77. doi: 10.3390/mps8040077 (PMC12286126; doi:10.3390/mps8040077)
Supplement: Supplementary file 1 [file mps-3708003 Ethics Commitee supplementary 4.pdf › Supplemetary files/mps-3708003 Ethics Commitee supplementary 4.pdf]

Local Ethics Research Committee
School of Physical Education and Sport Science at Serres
Aristotle University of Thessaloniki

Serres, 10-June-2025

To: Eleftherios Kellis, Professor

School of Physical Education and Sport Science at Serres,

Aristotle University of Thessaloniki, Greece

Subject: Decision on application for research protocol approval on ethics **(ERC-013/2025)**

Dear Dr. Kellis,

The ethics committee for research has considered for acceptance your application of the research project entitled “Effects of Core Training on Muscle Activation, Hypertrophy, Balance, and Quality of Life in Active Adults”.

The approval of this study is **under the condition** that all Authors complied with the Ethics Conduct in Research Involving Humans of the University and the EU personal data protection Act.

Based on the submitted documents, there are no ethical concerns and therefore the committee **approves** the submitted research protocol.

Please note that:

- all serious and unexpected adverse events should be reported to the committee as soon as possible for re-evaluation of the ethical approval decision.
- the committee approves the protocol and no changes are allowed, unless previously modified and approved.
- all research participants are to be provided with a Participant Consent Form, as supplied to the committee.
- copies of consent forms should be retained.
- personal data usage and storage policy will follow the General Data Protection Regulation requirements.
- As long as the experiments are held during the COVID-19 pandemic, the authors must fully comply with the guidelines issued by the National Public Health Organization (EODY) for the control/management of spread of COVID-19 throughout the experimental procedures (i.e., square meters per person, use of masks, social distancing, etc.)

The Committee wishes you success to this and all running projects.

X Andreas Zafeiridis 14.06.2025 06:16 UTC	X SYMEON VLACHOPOULOS 14/06/2025 09:35	X Dimitrios Patikas 13.06.2025 07:04 UTC
Zafeiridis A.<br>Professor	Vlachopoulos S.<br>Professor	Patikas D.<br>Professor
